# Mixed Reality-Based Monitoring and Supervisory Control of an Industrial Assembly Line Using Direct OPC UA and Modbus TCP Communication

**DOI:** 10.3390/s26196208

**Published:** 2026-09-30

**Authors:** Tudor Covrig, Adrian Duka, Liviu Miclea

**Affiliations:** 1 Automation Department, Technical University of Cluj-Napoca, 400027 Cluj-Napoca, Romania; tudor.covrig@umfst.ro; 2 Department of Electrical Engineering and Computers, University of Medicine, Pharmacy, Sciences and Technology of Târgu Mures, 540088 Târgu Mures, Romania; adrian.duka@umfst.ro

**Keywords:** mixed reality, industrial assembly line, OPC UA, Modbus TCP, industrial monitoring and control, industry 4.0

## Abstract

Supervisory monitoring and control systems require prompt access to process data and information. The systems described in the literature utilize Mixed Reality (MR) technology and have different system architectures and communication methods. Some systems require the presence of intermediary elements for data exchange between the MR environment and the industrial control system. In this article, we suggest and develop an MR-based system for the supervisory monitoring and control of an industrial assembly line. The uniqueness of the suggested system consists of bidirectional communication between the MR system and the main PLC using the OPC UA protocol without intermediary elements in the MR–PLC communication chain. The Modbus TCP protocol is used for obtaining parameters from the network analyzer. The system allows us to monitor some process parameters and sensors on the assembly line and transfer supervisory commands from the MR interface to the main PLC using virtual controls on physical elements. The system was built using the MRTK library inside the Unity environment. To experimentally evaluate the system, experiments were conducted on the industrial assembly line, where the process parameters and communication performance were recorded under experimental conditions.

## 1. Introduction

The new technologies implemented in industry involve the use of technical solutions consistent with the concept of Industry 4.0 [1,2]. They provide the capability to collect data, monitor processes, and perform supervisory control of technological processes. The tools used in industrial systems for these purposes include, among others, SCADA systems [3,4], HMI systems [5,6], and various other software applications. They allow users to access selected information collected from technological processes, while interaction usually takes place using traditional interfaces, which are independent of the surrounding physical environment of the industrial equipment. One technology that can be applied to develop systems for monitoring and supervisory control of technological processes is Mixed Reality (MR). Using MR allows virtual objects to be included in physical space, and users can interact with both physical and digital objects [7]. The literature review reveals that, in the studied industrial systems using MR, information related to the operation of equipment, processes, and operating procedures is provided [8]. Other implementations of these systems include the use of mechanisms for supervisory interaction with equipment and industrial objects. The solutions presented in the studied literature have architectures that include some intermediate elements, such as Digital Twins, cloud systems, databases, or additional servers, which may be used to process the data [9]. These intermediate elements have advantages such as interoperability, scalability, security, and data management. Nevertheless, if the objective is to investigate MR–PLC direct communication, the elimination of middleware from the communication path allows the communication characteristics between MR and the PLC to be investigated without extra communication layers. These solutions show how MR can be utilized in the field of integration of the digital world into the physical environment, allowing users to interact with technological processes [10].

While researching MR technologies presented in the literature, two fields of their application were identified. The first one refers to those systems in which MR is utilized as a tool for displaying information about process parameters and status information, providing instructions for the operation of equipment, but not allowing users to issue supervisory commands or influence the technological process. The second one relates to systems in which MR can be used for process monitoring and supervisory control; however, this feature can use some additional components for data and information processing, such as cloud systems, Digital Twins, or intermediate servers [11]. From this perspective, the research question addressed in the paper is the following: Is it feasible to implement industrial process monitoring and high-level command transmission to the PLC directly using MR communication, without any middleware along this communication path, and what are the communication properties of this configuration in the considered experimental environment? In this case, it is possible to investigate the communication between the MR application and the PLC directly and analyze such parameters as response times and communication variability without adding additional communication layers. In other words, the goal of this paper is not to consider any middleware as undesirable, but rather to investigate the feasibility and the properties of the communication between MR and the PLC in the direct MR–PLC configuration. To solve the problem stated above, this work presents the design, implementation, and experimental evaluation of an MR-based system for monitoring and high-level supervisory control of an industrial assembly line. The main scientific novelty of this paper can be found in the experimental investigation of the feasibility of the MR–PLC architecture with direct bidirectional communication, as well as in the experimental characterization of its communication properties under the specified conditions. Bidirectional communication is carried out using OPC UA without any middleware in this communication path. The Modbus TCP protocol is additionally applied for obtaining parameters from the network analyzer. Hence, the main novelty of this paper is not only the implementation of the system, but also the experimental investigation of the direct MR–PLC communication and the measurement of its communication properties in an industrial environment. The system provides the acquisition and processing of selected process data and information from the industrial assembly line. Low-level control functions in the industrial line are implemented in the PLC, while the MR system is used for process monitoring, configuration, and transmission of high-level supervisory commands.

## 2. State of the Art

Monitoring and supervisory control systems employed in industrial processes make use of different infrastructures, such as SCADA systems, web technologies, and software platforms. Regarding Industry 4.0, monitoring and supervisory control systems utilize connected devices, industrial communication networks, and data-based technologies to facilitate process monitoring, supervision, and interaction with operators. Monitoring and supervisory control systems in industrial facilities continue to be widely applied using SCADA and HMI systems. One technology that could be employed to design and implement such systems is Mixed Reality (MR). Mixed reality involves the superposition of virtual elements onto the physical world, so that it becomes possible to interact with both virtual and physical elements [12]. Mixed reality combines concepts from virtual reality and augmented reality. In virtual reality, users interact mostly with virtual objects in a computer-simulated environment, while in augmented reality, virtual objects are superposed onto the real environment and help users access and interpret information [13,14]. Therefore, mixed reality can be seen as an expansion of technologies related to virtual and augmented realities, since it allows the integration of virtual objects into the real world and interaction with both virtual and real-world entities.

The reviewed papers describe systems that make use of mixed reality in industrial processes to perform different activities, such as information visualization, process monitoring, and interaction with industrial devices. Paper [15] introduces a system in which process data is gathered from an industrial system and then visualized via a mixed reality interface. Technological process simulation occurs in the mixed reality environment, whereas the link between the mixed reality system and the industrial process is created via a cloud system. Data is collected via different sensors. Paper [16] introduces a system for predictive maintenance and data visualization via a mixed reality interface. It is possible to observe both physical equipment and the data related to it at the same time, and the technology uses IoT, Digital Twin, artificial intelligence, and mixed reality technologies. Paper [17] introduces a system for real-time monitoring of industrial assembly lines. Mixed reality is used for displaying process information and for monitoring the technological process. Systems with a combination of mixed reality and augmented reality are used for real-time monitoring of processes and data and for providing data to users during the manufacturing process [18,19]. Moreover, there are systems using mixed reality for assisting operators in the manufacturing process [20]. Integration of XR technologies in industrial environments was also studied in terms of their integration with Industry 4.0 technologies (IoT, Digital Twin) [21,22]. In other papers, Edge Computing and Computer Vision technologies are used in addition to AR/VR technologies to perform process monitoring, control-oriented tasks, and data processing [23,24,25,26]. While these architectures might offer additional capabilities for data processing and management, depending on the chosen architecture, additional elements might be required in the data communication and processing pipeline [27,28,29,30,31]. Moreover, some of the considered approaches apply predefined communication schemes and additional infrastructure to help with data exchange between the application and industrial devices [32,33,34]. The papers mentioned demonstrate the creation of systems using XR technologies for interaction with industrial devices and real-time visualization of process information. However, these systems utilize different communication schemes, offering varying functionality from data visualization and monitoring to command transmission to industrial devices [35,36]. For the comparison of these architectures, criteria such as architectural and functional aspects were evaluated, including the XR technology used, the industrial field of application, the communication protocols used, the availability of intermediate components, the direction of communication, monitoring capabilities, transmission of high-level commands, and the location of data processing. Such criteria are used for characterizing the analyzed solutions, but not for creating a performance hierarchy, because various architectures may have their own advantages and drawbacks. Based on the literature review, existing solutions differ in terms of communication architecture, availability of intermediate components, and the interaction level of the XR solution with industrial equipment. In the scope of the analysis of existing literature, there is no solution which combines, within a single XR solution, direct bidirectional communication with a PLC, process monitoring, and the transmission of high-level commands to the control system. This conclusion is drawn solely based on the selected studies for analysis and should thus be viewed in the light of the limitations of the criteria for the selection of the studies and the scope of the literature used. This is the aspect that this system addresses in this paper.

In this study, process control is differentiated from supervisory functions. Low-level process control, which encompasses sequence control and direct control of process equipment, is performed using the PLC. The mixed reality system supports the functionality required to monitor the status of the process, visualize process parameters, and issue high-level commands to the PLC. The bidirectional communication consists of the MR application communicating process data and status, as well as issuing high-level commands from the MR application to the PLC. Thus, in the current case, the transmission of high-level commands from the MR application to the control system represents an example of supervisory control without the MR application taking the place of the low-level control carried out by the PLC. To emphasize the difference between supervisory and process control throughout the text, the concept of supervisory control is used to characterize the process of transmitting high-level commands from the MR application to the control system, while the concept of process control is used to describe the control performed by the PLC. A comparison in Table 1 is made among some of the systems using either mixed reality or augmented reality, and the system described in this paper. It should be noted that the list of selected articles is not intended to be a full review of all the literature on the topic but rather includes articles that are representative of the main approaches found among the reviewed studies. To make the comparison, papers were chosen in which the authors describe systems with functionalities based on mixed reality or augmented reality technologies used in industrial processes, giving descriptions of their architecture, interaction with equipment, monitoring, interaction with industrial equipment, and information management technologies used. The criterion related to direct communication with the PLC refers to the direct communication of data between the MR application and the PLC without any intermediate communication devices. This means that the communication channel between the MR application and the PLC is devoid of any software/hardware components that facilitate the communication between the systems. The criterion of “supervisory command transmission” implies that the mixed reality application can transmit supervisory commands, but the execution of control logic is the responsibility of the PLC. The proposed system provides a direct connection between the mixed reality application and the equipment of the industrial line. For transferring data through this direct connection, the OPC UA and Modbus TCP communication protocols are used. In the proposed system, the MR–PLC connection is used not only for reading process data but also for sending high-level commands to the PLC. Node-RED and the database in the architecture of the proposed system are not part of the MR–PLC communication path that is used for monitoring and supervisory purposes. These components perform functions such as collecting, processing, and analyzing the required process data and information from the industrial assembly line without participating in communication between the MR system and the equipment of the industrial line. Thus, the presence of Node-RED and the database in the architecture of the system does not contradict the direct MR–PLC communication, since these components are not intermediate elements in the process of data transfer. Considering these criteria, Table 1 compares the systems described in the existing literature with the system described in this paper.

## 3. System Design

### 3.1. System Objectives

The purpose of this paper is the development and evaluation of a system for supervisory monitoring of the operation of an industrial assembly line. The developed system involves the application of MR technologies to create an interface for process monitoring and supervisory control. The methodology for this research comprises the integration of development and validation stages in the experimental research conducted on the industrial assembly line. The aim of the research is the investigation of the application of MR for process monitoring, data visualization, and the transmission of supervisory commands to the industrial process. The system design is based on the analysis of the operational and monitoring requirements of the industrial assembly line concerning process state monitoring, the acquisition and visualization of process-related data and parameters, process state interpretation, user notification and supervision, and supervisory command transmission. Based on this, the system architecture is defined, and the responsibilities of the hardware and software are specified. In accordance with the proposed system architecture, supervisory process monitoring and user interaction via the mixed reality application are separated from the low-level automatic process control functions. Low-level control functions and process execution are carried out by the main PLC of the industrial assembly line. The mixed reality system performs process monitoring, parameter configuration, and supervisory command transmission to the process, but does not interfere with the low-level control of the process. Consequently, the mixed reality application provides user interaction with and monitoring of the process at the supervisory level, while maintaining the process control automation performed by the PLC. The requirements formulated for the purposes of status monitoring, data gathering and visualization, user notification, and the transmission of supervisory commands are reviewed in the context of the experiments conducted in the validation part of the paper. The experiments evaluate MR–PLC communication, process status monitoring, the transmission of supervisory commands, and the acquisition and visualization of selected process data. These experiments are intended to verify the implementation of the formulated system requirements and their corresponding functionality under the experimental conditions investigated.

### 3.2. Operational Requirements

The specification that formed the basis for the design of this system is one that provides real-time monitoring of the assembly process and supervisory-level interaction with the production line. The primary specifications of the system include process parameter monitoring, display of process status information, part configuration selection, transmission of supervisory commands to the PLC, and detection of operational errors. Sequential and automatic control of the machinery on the production line is carried out using the main PLC. One significant element of the system architecture is the connection between the MR system and the main PLC via direct communication. This implies the exchange of data and transmission of supervisory commands from the mixed reality system to the PLC without the use of Node-RED or the database. Node-RED and the database are used independently for data collection, storage, and analysis, without interfering with the MR–PLC communication used for monitoring and supervisory control.

From the MR application standpoint, system status information is evaluated for the purposes of presenting the state of the process and its parameters, determining the necessity of operator intervention, and generating alerts. The MR application can transmit supervisory commands to the PLC, whereas sequential control and critical process-control functions are performed by the PLC-based control system. Thus, the MR application acts as an interface for monitoring and supervisory interaction and does not perform low-level control tasks. The main limitations of the system include wireless communication, hardware limitations of the HoloLens 2 device, and the requirement to maintain critical process control functionality in the existing PLC-based control system. The industrial assembly line is intended to work as a distributed automation system that performs assembly processes across different stations. Operation of the system is based on the sequential execution of the assembly processes, recognition and transfer of selected configurations from one station to another, proper positioning of parts, synchronization of stations, and equipment monitoring. This allows the integration of the mixed-reality system as a monitoring and supervisory control interface without replacing the existing PLC-based control system. Data collection and storage are performed separately using a Node-RED workflow and the database.

### 3.3. Description of the Production Line and Assembly Process

This section describes how an industrial assembly line can be used to assemble parts. The industrial assembly line is in a laboratory within the School of Engineering and Information Technology. The industrial assembly line comprises five stations where the parts are assembled using the selected configuration specified by the user. The figure below illustrates the structure of the industrial assembly line.

**Figure 1:**
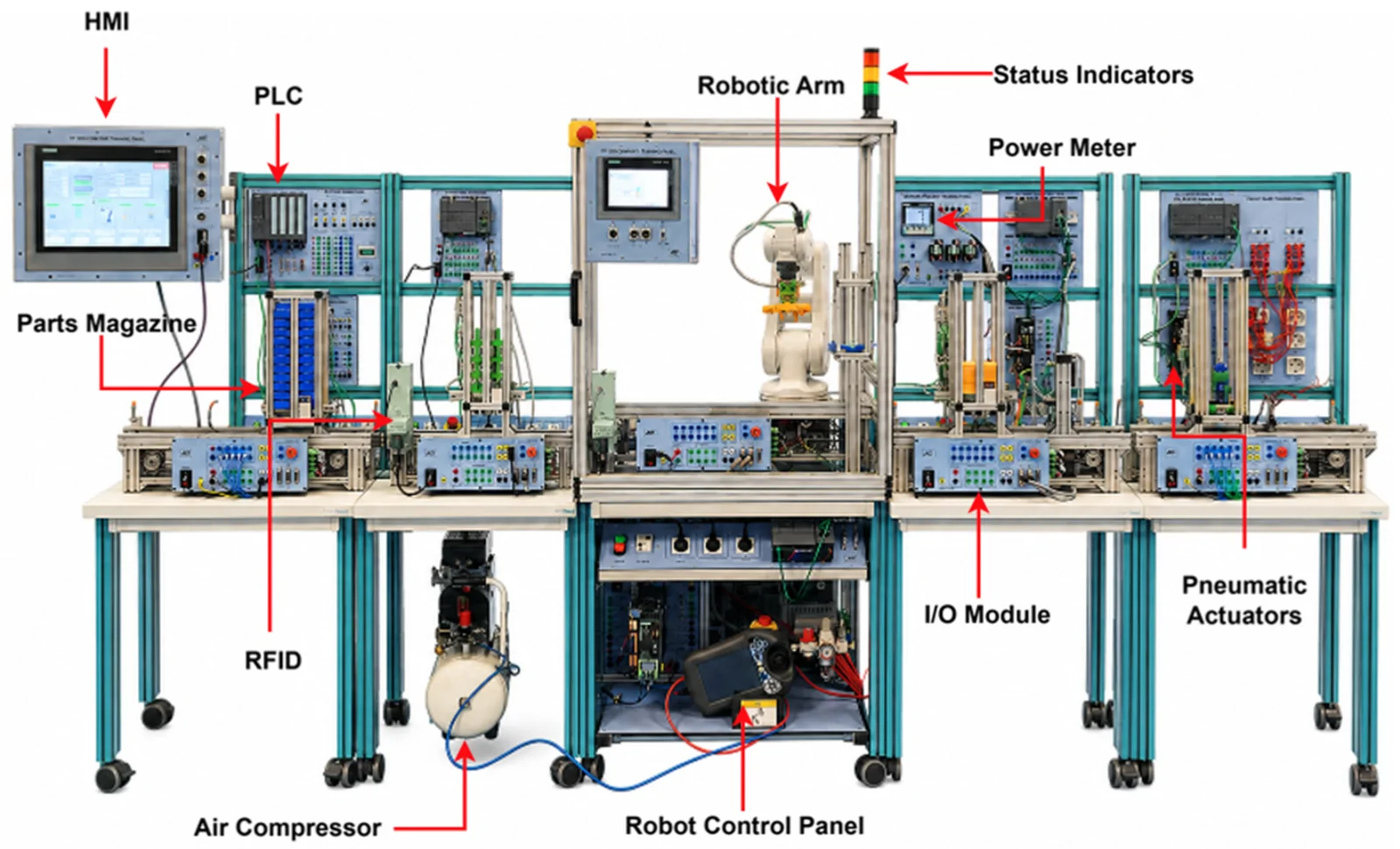
Industrial assembly line.

The industrial assembly line is composed of five stations, as illustrated in Figure 1. The hardware components used for the assembly processes at these stations include PLCs of the S7-1200 and S7-1500 series, TP 700 Comfort and TP 1500 Comfort HMIs, frequency converters, and RFID equipment (Siemens, Munich, Germany). The use of several PLCs and HMIs results from the distributed nature of the assembly line, where different stations have different roles related to the control of equipment. Station 1 has a PLC that acts as the main PLC of the entire assembly line and controls the assembly processes and communication with other stations and equipment. The other PLCs are used to control the equipment located at their respective stations. The HMIs allow operators to interact with the equipment locally but are not involved in communication between the mixed reality system and the main PLC. The positioning of the rectangular parts is performed using an ABB IRB 120 robotic arm (ABB, Zurich, Switzerland), which is installed at Station 3. The movement of the robotic arm is managed by the PLC-based automation system. The mixed reality system provides supervisory monitoring and interaction with the assembly process but does not perform or substitute for any low-level control functions of the robotic arm.

**Figure 2:**
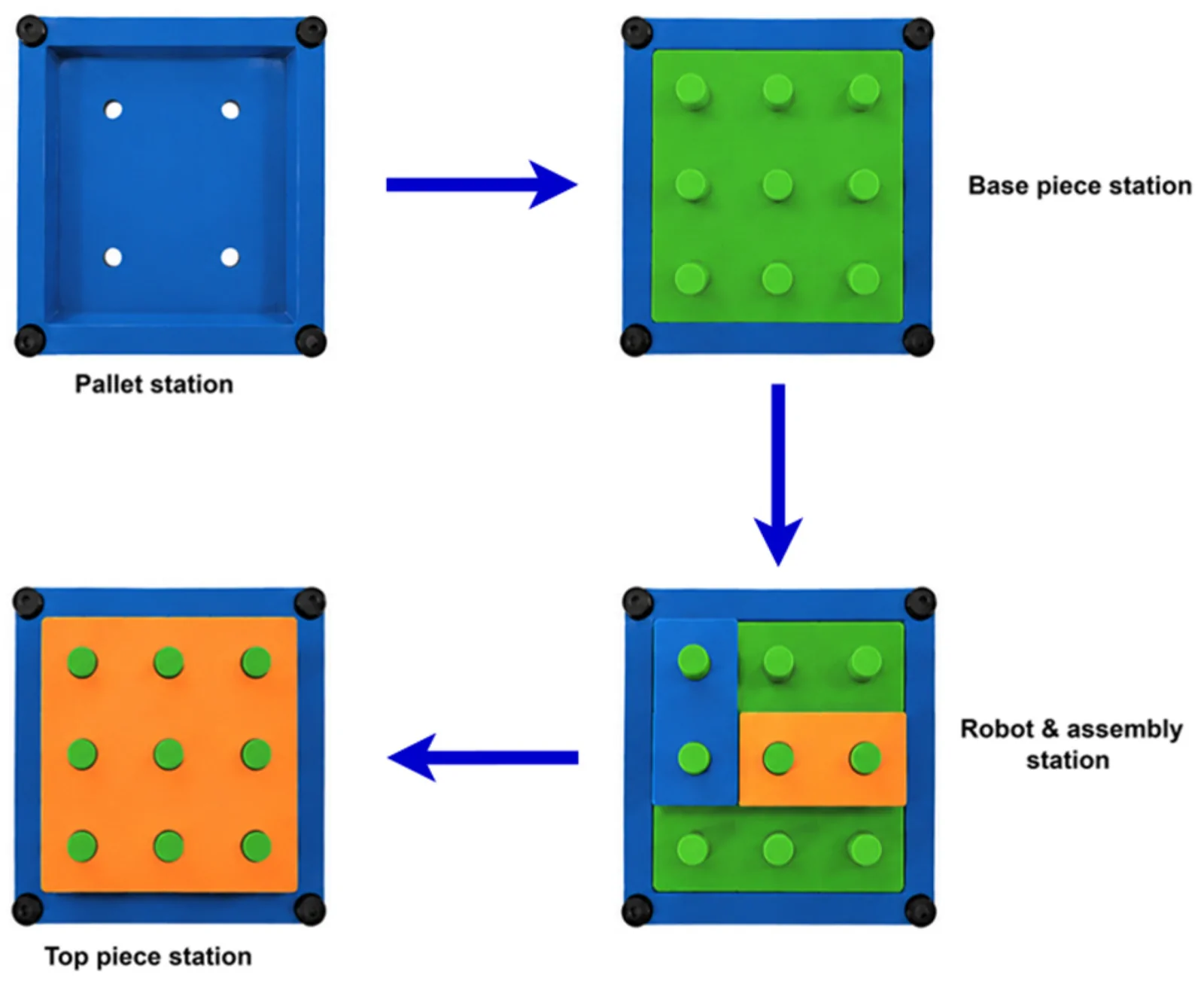
Steps for Assembling the Parts.

The industrial assembly line consists of five PLCs and two HMIs. The main PLC controls the execution sequence of the assembly process, whereas the remaining PLCs manage the assembly activities at their respective stations. Two HMI interfaces are used for local operator interaction with the equipment. The mixed reality system does not substitute for the functions of these interfaces; rather, it complements their capabilities. The finished assembly comprises four main components. The first component includes the RFID tag, which is used to identify the product and provide position data for the subsequent stations that utilize this information in the assembly process. The second component acts as the base on which the robot at Station 3 places the relevant parts based on the position data received from Station 1. The last component of the assembly is the orange component, which is used for fixing the other components together. The assembly process is demonstrated in Figure 2.

**Figure 3:**
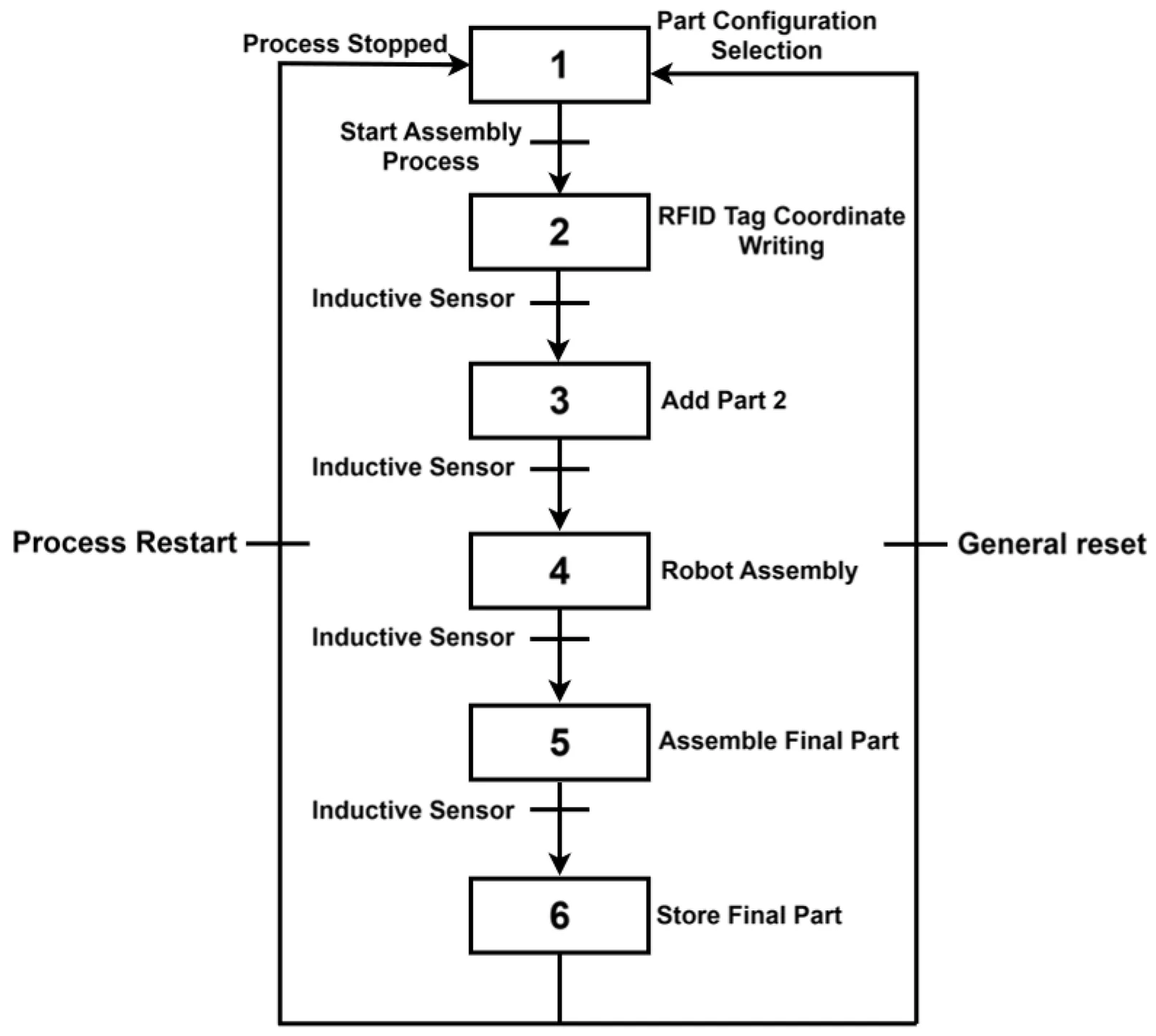
Operational Stages of the Industrial Line.

The assembly process performed by the production line comprises several steps. The first step concerns the selection of the parts configuration. This is achieved using a mixed reality system, through which information about the selected parts configuration is transmitted as high-level supervisory information to the main PLC. After this step is completed, the production line begins the assembly process. The second step is performed using RFID equipment, which is used to determine the coordinates chosen by the user. The third stage is associated with the placement of the second part. In the fourth step, the robot places the rectangular parts by using the coordinates obtained from the RFID tag. The fifth stage involves assembling the last part, the orange component, which secures all other parts. The last stage involves storing the assembled part after its assembly. With the given configuration, the assembly process is executed using the above-mentioned parts. The RFID system coordinates the positioning of parts, while the configuration is selected using the mixed reality system and the assembly is performed by the equipment of the industrial line. Inductive sensors are used to indicate the transition between stations and, consequently, between the different assembly steps. The whole process can be halted and restarted at any point of operation using the general reset function. The structure of the stages of operation for the industrial assembly process is illustrated in Figure 3.

### 3.4. Architecture of the Monitoring and Control System

**Figure 4:**
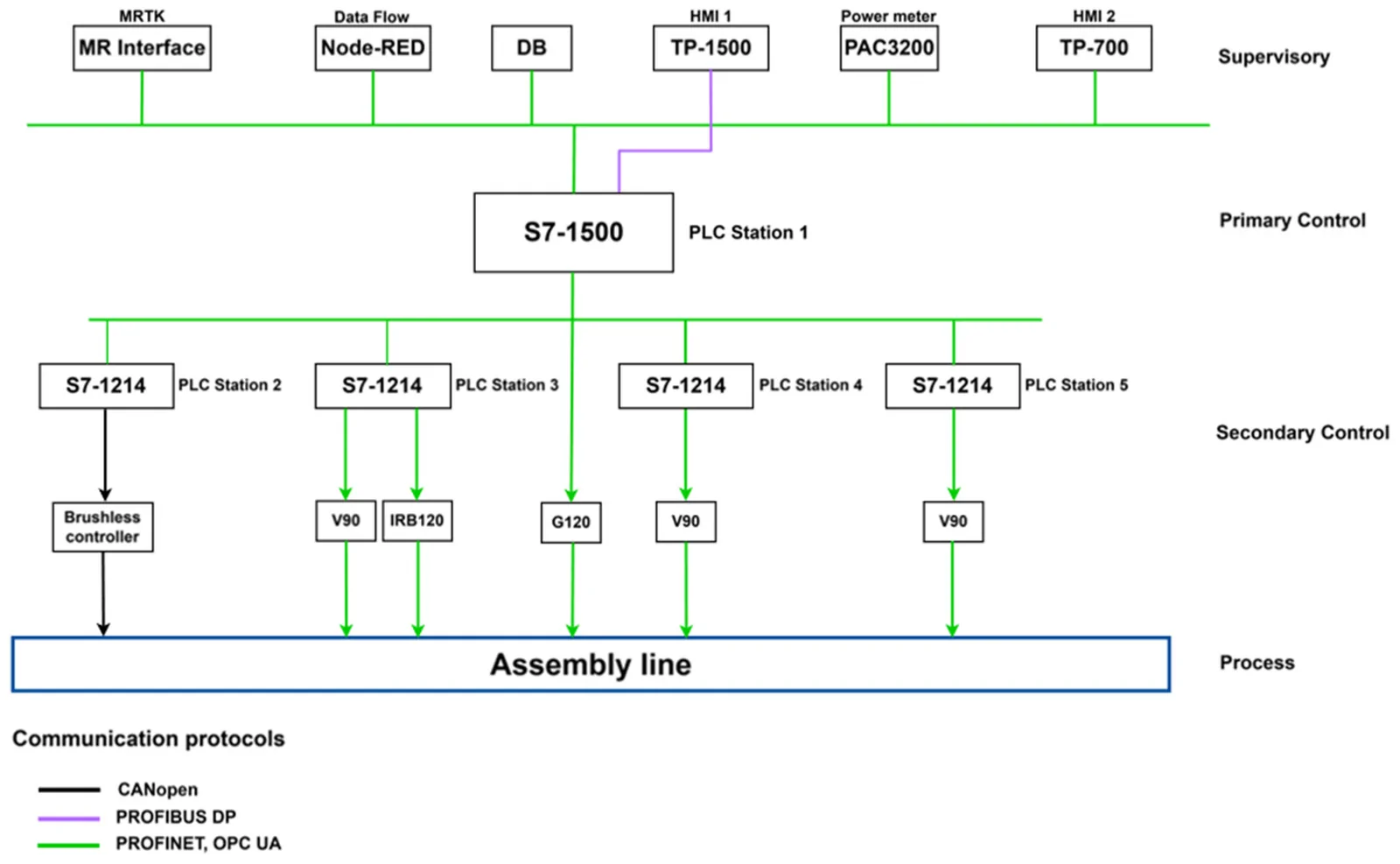
System operation diagram.

The architecture of the system consists of numerous hardware and software elements, arranged across several levels of operation. The first of these levels is the supervision level, which includes elements related to process monitoring and management, data management, and supervisory interaction with the industrial process. The key element of this level is the Mixed Reality (MR) system. In the framework of the proposed system architecture, the MR system is used for process monitoring and supervisory interaction with the industrial process, while sequential and low-level equipment control is performed by the PLC-based automation system. Data management and visualization are provided by other supervision-level elements, namely HMIs, the database, the network analyzer, and Node-RED. These interfaces and the database do not provide communication between the MR system and the main PLC but are used only to collect and analyze data regarding the manufacturing process. The next level includes the main PLC control layer, in which the assembly process is carried out. The main PLC organizes the process and ensures sequential performance of the assembly process, exchanges data between the MR system and other elements of the assembly line and sends control signals to the equipment at the stations. Therefore, the communication channel used for real-time monitoring and transmission of supervisory commands operates directly between the MR system and the main PLC, without Node-RED and the database acting as middleware. The third layer is the station control system, and it comprises four PLCs that control station-specific equipment, such as the frequency converter and the RFID reader. The fourth and final layer of the operational architecture is the industrial process layer, which involves physical processes and equipment, including pneumatic machines and conveyor belts, among others, used for the assembly and transfer of parts between stations. The whole operational architecture is shown in Figure 4.

Two different data flows are shown in Figure 4. The first data flow involves real-time monitoring and supervisory control, which are performed directly through the mixed reality system and the main PLC. The second data flow involves data collection and storage, in which data is transmitted to the Node-RED interface and the database for further analysis. As shown in Figure 4, Node-RED and the database are not intermediary systems for the transmission of data between the mixed reality system and the PLC.

### 3.5. Mixed Reality System

The solution proposed in this paper is based on the use of mixed reality technology that allows the user to interact with the industrial assembly process. This solution is developed with the help of the Unity platform, since this platform allows the development of mixed reality applications and the integration of the HoloLens 2 mixed reality headset with the MRTK framework. Moreover, the Unity platform provides possibilities to interact with virtual objects overlaid on the industrial process. The MRTK framework provides the foundation for the development of the mixed reality interface and provides the functionalities needed for real-time interaction with virtual objects. The HoloLens 2 mixed reality headset is used to visualize the solution and to overlay virtual objects on the physical parts of the assembly line.

The solution provides live data related to selected operational parameters and the status of the industrial assembly line. The proposed solution offers capabilities for process configuration, parameterization, monitoring of operational modes, and detection of operational modes requiring user attention. Process status is established based on the data obtained from the PLC and network analyzer. The obtained data is further processed by the mixed reality system to establish the current state of the process and provide the necessary information to the user. Supervisory-level commands can be generated and sent to the PLC based on the current state of the process and user actions. The measured variables include electrical values and information about sensors connected to the industrial assembly line. Low-level control and process control logic are realized within the PLC at Station 1. Hence, the mixed reality system does not carry out any real-time low-level control of the industrial process and does not substitute for control or safety-critical functions realized through the PLC. Rather, the mixed reality system performs supervisory-level monitoring, configuration, parameterization, and command transmission. The key limitations of the system are its reliance on wireless communication and the restrictions imposed by the field of view of the mixed reality headset. The mixed reality system allows for the configuration and positioning of components, but it does not provide for their replacement. Replacing the set of components will require changes to the control logic of the stations and to the assembly sequence implemented in the PLCs.

### 3.6. Communication Architecture and Data Flow

Communication between the elements of the system is ensured by several communication protocols. Communication is carried out by means of the PROFINET protocol among most of the system elements. The HMI at Station 1 is connected to the main PLC through the PROFIBUS protocol. Communication between the PLC at Station 2 and the brushless motor is provided by means of the CANopen protocol. The OPC UA protocol is used for communication between the mixed reality system and the main PLC. The library that provides this communication is Opc.UaFx.Advanced. It is included in the Unity project with the help of the NuGet Package Manager. This implementation enables direct communication between the mixed reality system and the PLC at Station 1. In the current architecture, OPC UA serves as the direct channel for the exchange of data between the mixed reality system and the main PLC, whereas Modbus TCP is responsible for the communication between the mixed reality system and the network analyzer. The mixed reality system acquires the necessary process data and processes it to obtain relevant information about the process. The commands from the user are also transmitted through OPC UA, which forwards the commands directly to the PLC. These commands are supervisory-level commands rather than low-level control commands. The data flow can be described as follows: first, the mixed reality system retrieves the selected data from the industrial assembly line via communication interfaces; second, the communication availability is verified; third, the selected data are processed; fourth, the current process state is defined; and fifth, the information is provided via the mixed reality interface. Once a command is issued through the mixed reality interface, it is forwarded to the main PLC as a supervisory command. The control operations and control logic remain implemented within the PLC. The data concerning the status of the industrial assembly line is retrieved from the network analyzer and processed in real-time to be displayed through the mixed reality interface.

**Figure 5:**
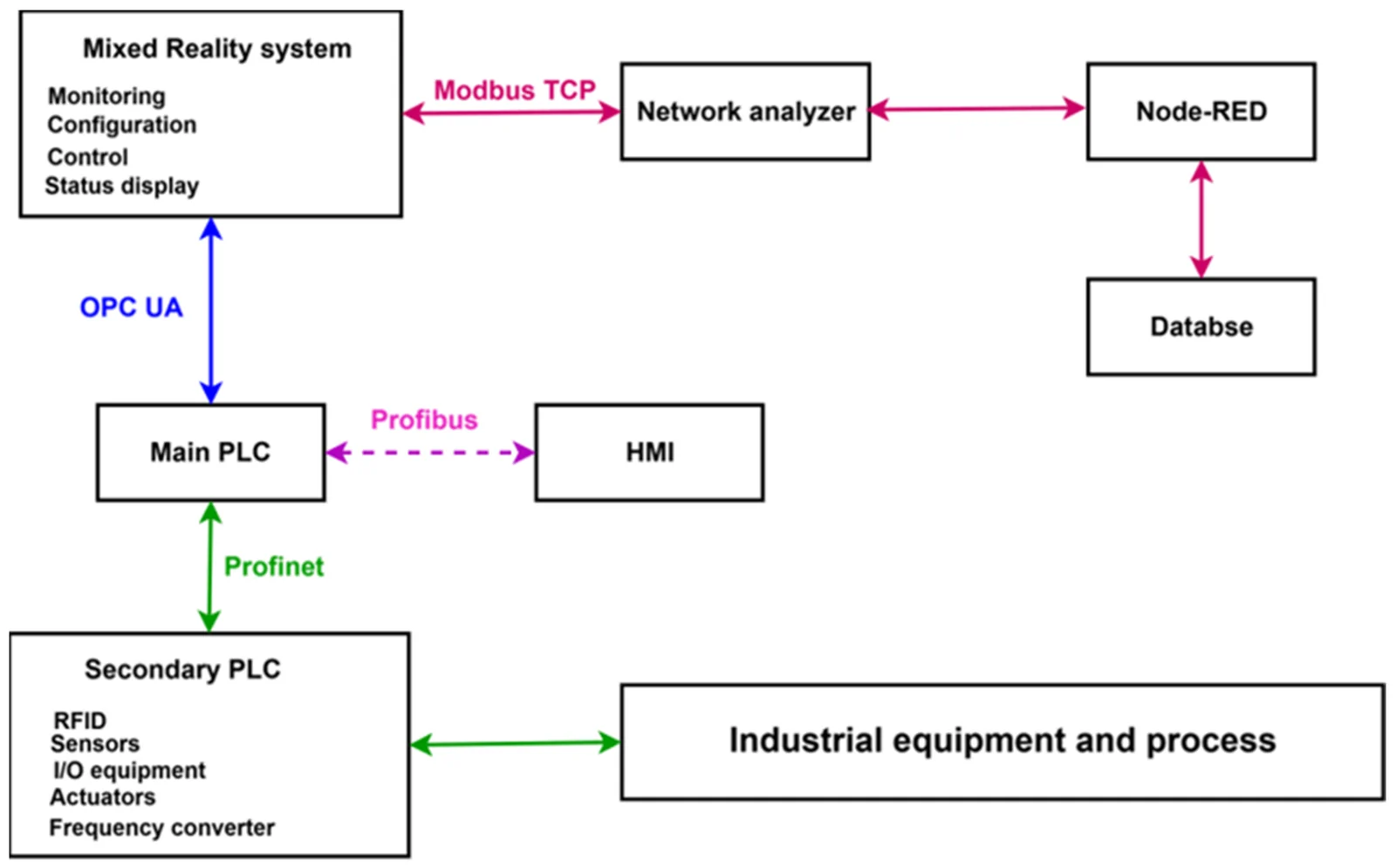
Communication Architecture and Data Flow.

The database serves the purpose of storing data and supporting further analysis. Data is transmitted to the database via the Node-RED interface. The above-mentioned data transmission path does not form part of the direct MR–PLC communication path and, therefore, is not used during the supervisory interaction between the mixed reality system and the PLC. Wireshark is used to analyze network traffic and identify potential communication errors or network-related issues by analyzing the data traffic between the system components. The database serves the purpose of storing data and supporting further analysis (Figure 5).

### 3.7. Data Processing and High-Level Order Management

In the mixed reality system, data acquisition, processing, and presentation take place via different processes. Initialization of the system’s components is performed in the first process. Two different protocols, namely OPC UA and Modbus TCP, are employed for the acquisition of data from the PLC and the network analyzer, respectively. In both cases, communication and configuration of the necessary parameters are performed to establish a successful communication connection. When communication is successfully established, the mixed reality system acquires the selected data from the industrial assembly line. This is followed by data processing before the data are displayed or the current process state is determined. Based on the identified process state, supervisory monitoring takes place, which includes visualizing the process data and providing notifications to the user. When the system is running normally, the data is updated within the mixed reality environment. In the case of an order issued by the user, the mixed reality system sends the respective supervisory command to the PLC, which processes the command according to the implemented control logic. In the case of an error, the mixed reality system informs the user, while control and shutdown procedures according to the automation logic are processed independently by the PLC and the industrial equipment. In the case of communication loss, the system pauses data processing until communication is restored, and the related error is displayed in the console. The detection of anomalies in the mixed reality system is used only for monitoring and user notification purposes and does not represent a safety procedure. Automated shutdowns that are part of the control logic and safety functions are independent of the mixed reality system and are performed at the PLC and industrial equipment levels. Reading and writing processes are independent and are not carried out constantly or concurrently. The reading procedure is necessary for acquiring monitoring data, while the writing procedure is performed only when it is necessary to send commands or configuration parameters to the PLC. Even though both procedures are part of the common workflow of the system, they are triggered for different purposes. For the SENTRON PAC3200 (Siemens, Munich, Germany) network analyzer, the mixed reality system communicates with the analyzer via the Modbus TCP protocol on a read-only basis and does not send written requests to the analyzer. To communicate with the analyzer, the EasyModbus library is used in the Unity application. Modbus TCP is not secure out of the box, nor does it offer encryption, and the EasyModbus library used in this implementation does not implement TLS-based encryption out of the box. Security is therefore implemented mainly at the infrastructure level and consists of network isolation and restricted access to the Modbus TCP communication port. Network analyzer access can be limited to authorized devices only, thus limiting access to the data being transmitted. In addition, the PAC3200 analyzer also implements PIN code protection for the device parameters, which limits access to the device settings but does not affect Modbus TCP communication security.

**Figure 6:**
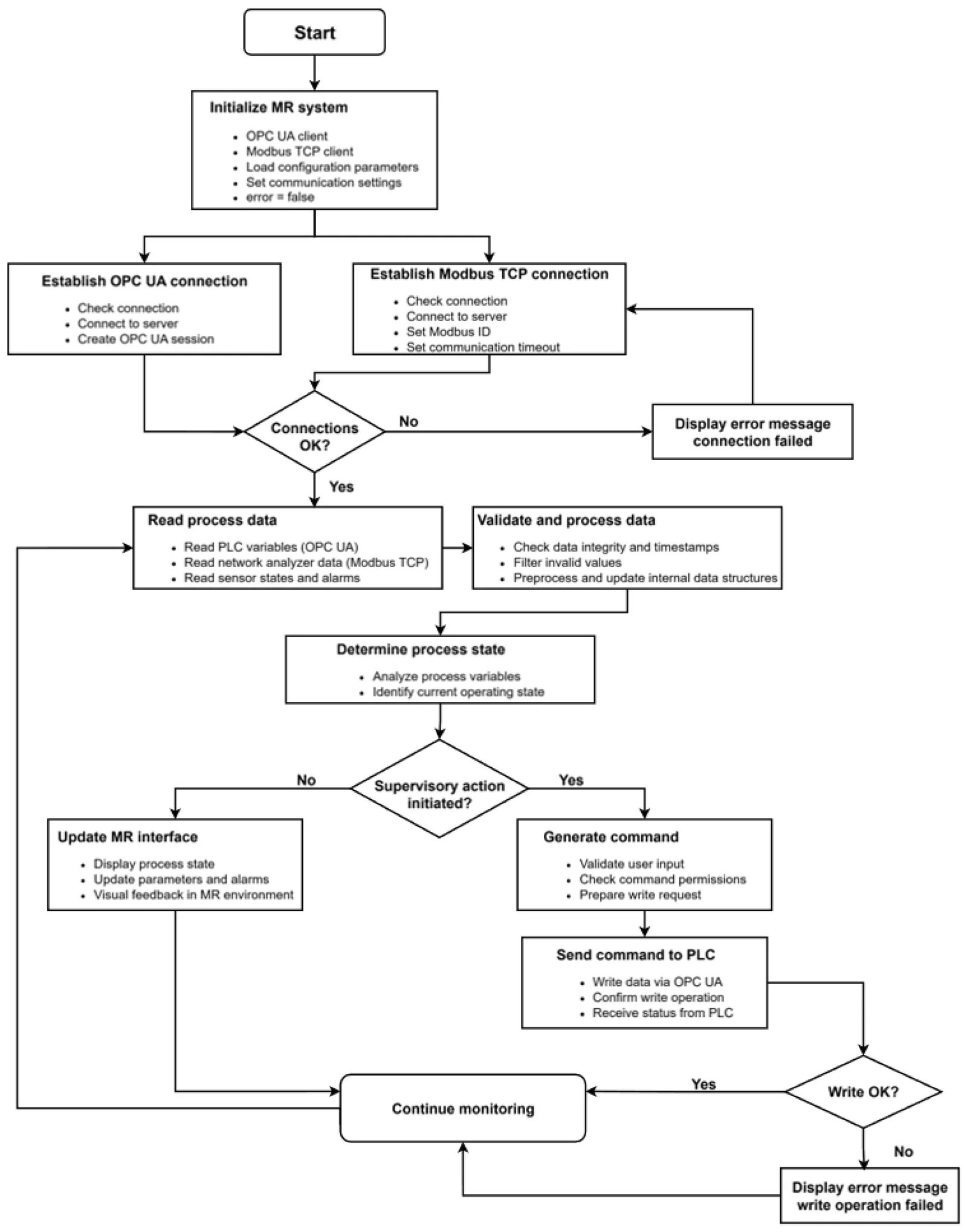
Data Processing Diagram.

In the case of OPC UA communication, security is implemented at the PLC level and uses digital certificates for the OPC UA service. This mixed reality system allows process monitoring and the transmission of supervisory-level commands to the industrial assembly line. The commands that can be issued using the mixed reality interface are limited to supervisory commands and do not replace or modify the independent control and safety functions of the industrial line. The safety functions are carried out by the control and industrial systems and are not dependent on the presence or operation of the mixed reality interface or the OPC UA communication interface. The mixed reality interface uses the console to show the communication status of the PLC and the network analyzer, including status information and error messages during system operation, as shown in Figure 6.

## 4. System Implementation

### 4.1. Components Used

The mixed reality application has been created within the Unity 6.2 development environment by means of the Mixed Reality Toolkit (MRTK), which provided the interface and interaction mechanism for virtual objects. The mixed reality application was built to run on the Microsoft HoloLens 2 platform, and the software tools were chosen considering their ability to integrate the mixed reality interface with industrial automation systems. In the Unity development project, MRTK offers the components that are needed to render and position virtual objects and provide interaction with the interface. To integrate the application with industrial systems, .NET components from NuGet packages were used.

**Figure 7:**
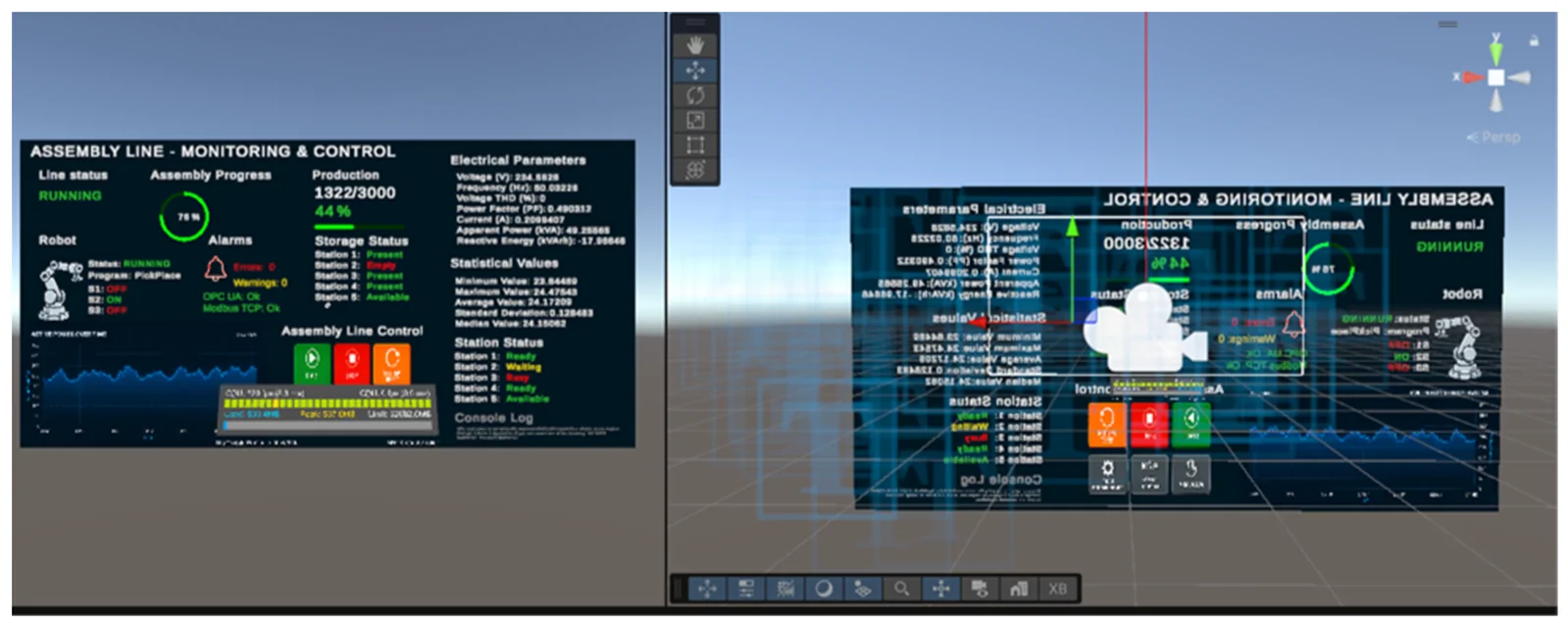
Structure of Software Components Used.

Thus, the software implementation is organized into three major layers: (1) Unity/MRTK for the mixed reality interface and interaction, (2) .NET libraries and NuGet packages for communication and data processing, and (3) HoloLens 2 for executing the application. This organization ensures the separation of functions related to the interface, communication, and processing of data. The structure of the software components implemented within the proposed system is presented in Figure 7.

**Figure 8:**
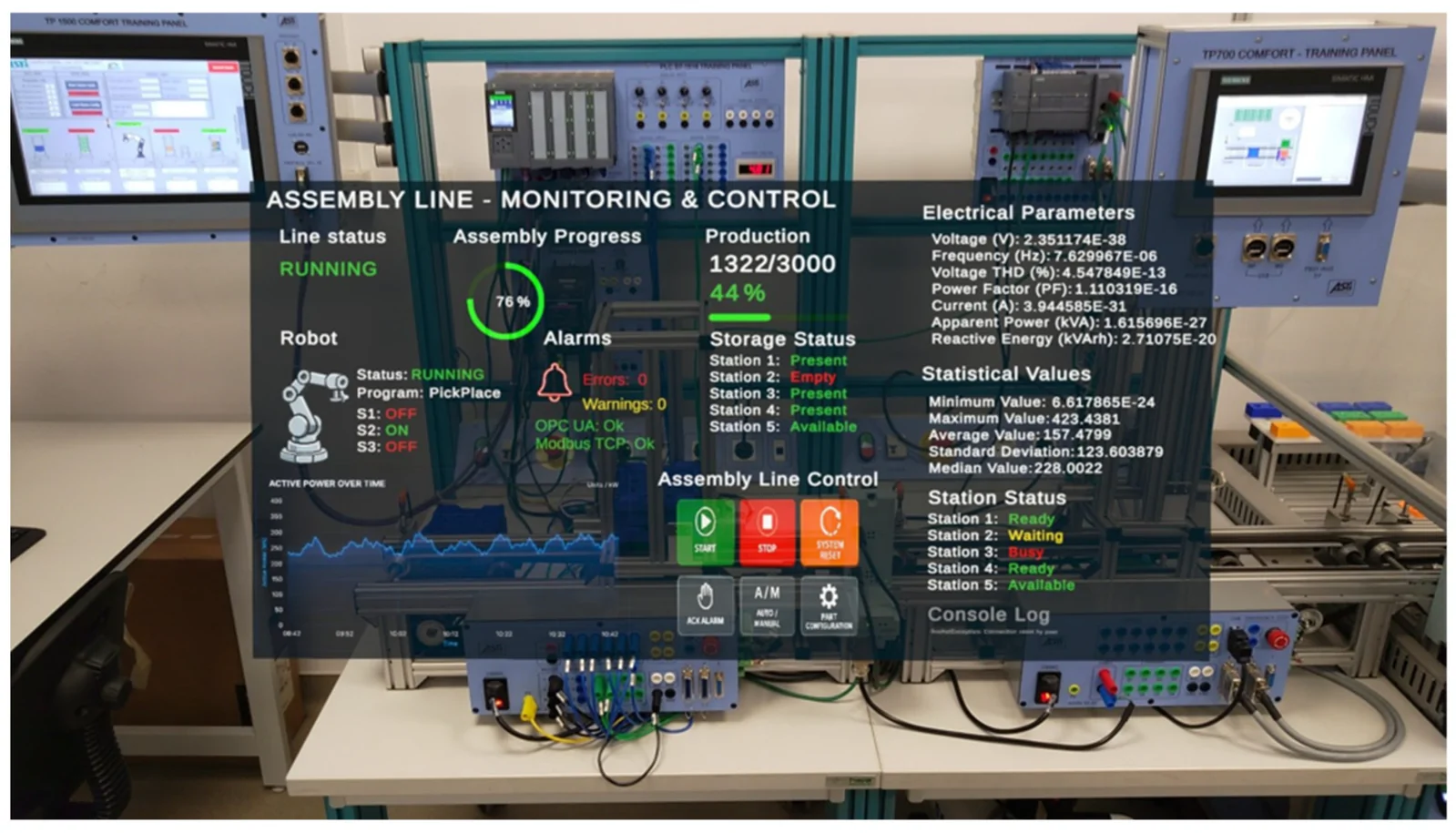
MR Interface for Monitoring and Control.

As depicted in Figure 7, the interface comprises several elements used to display and interact with data and information. These elements include text fields, sliders, panels, canvases, and buttons. They support the presentation and interaction with information exchanged between the mixed reality application and the industrial systems. The developed interface supports the monitoring and supervisory control of the industrial assembly line. The structure of the interface during operation using the Microsoft HoloLens 2 mixed reality headset is illustrated in Figure 8.

The monitoring and supervisory control interface of the mixed reality application is shown in Figure 8, illustrating how process information is presented to the operator through the HoloLens 2 device. The interface includes information on the state of the assembly line, the connection status of the physical equipment, the state of each workstation, process progress, the planned production volume, electrical parameter values, and statistical information. The monitoring functionality is illustrated for Station 3, which is equipped with a robotic arm and three inductive sensors. The data from the sensors is processed by the automation system, which determines the part position and executes the required process cycle. Information on the state of the sensors and the operating status of the robot is obtained by the MR application, while low-level control and process execution are handled by the PLC. The other stations on the assembly line can also be monitored through the interface. Virtual gauges are used for displaying process progress and production volumes, while the status of the stations is displayed using dedicated status indicators. The console log contains information on operational messages and errors and is used for monitoring the application status during operation. The assembly line control panel allows the operator to execute supervisory commands, such as starting and stopping the process, resetting the process, and configuring certain parameters concerning individual components. Supervisory commands are executed by the PLC, while the process sequence and low-level control logic are programmed into the main PLC of the assembly line. Thus, the MR application acts as a supervisory monitoring and control interface, while the PLC remains responsible for executing the underlying control logic.

### 4.2. Communication with the Industrial Assembly Line

Two protocols are used to communicate between the industrial line and the mixed reality application to transfer data. The choice of communication protocol depends on the type of data acquired from the industrial line. In particular, the OPC UA protocol is used to transfer information about the status and supervisory control of the industrial line, as well as supervisory commands sent to the main PLC through this protocol. The Modbus TCP protocol is used to obtain electrical parameter data from the SENTRON PAC3200 network analyzer. In the MR application, the following libraries are used: Opc.UaFx.Advanced for the OPC UA protocol and EasyModbus for the Modbus TCP protocol. It is important to note that communication takes place directly between the MR application and the PLC without the use of an intermediary (Node-RED or the database). Communication between the MR application and the SENTRON PAC3200 network analyzer also takes place directly, using the Modbus TCP protocol. The software-configured time interval between consecutive data acquisition cycles is 10 ms. The 10 ms period corresponds to a nominal setting of 100 Hz but does not represent an experimentally verified continuous acquisition rate or a PLC control cycle. Therefore, the 10 ms period should be understood as the configured acquisition interval of the MR application rather than as a measured communication or control frequency. The workflow of the developed application is presented in Algorithm 1.
**Algorithm 1:** System Connection**1. Given:** OPC UA server, Modbus TCP server, error text field **2. Initialize:** opcClient= new OpcClient(“opc.tcp://192.168.1.5:4840”)         modbusClient=new ModbusClient(“192.168.1.42”, 502)        error = false **3. function Start** **4.    try** **5.**      opcClient.Connect() **6.**      modbusClient.Connect() **7.**         Log(“Industrial devices connected”) **8.**           error.text ← “Industrial devices connected” **9.      catch** Exception e **10.**          error ← true **11.**          Log(“Failed to connect: “ + e.Message) **12.**          error.text ← “Failed to connect: “ + e.Message **13.      end try** **14.      return error** **15. end function**

The initialization process is aimed at establishing and verifying the connections between the MR application and the industrial machinery using the OPC UA and Modbus TCP protocols. During initialization, the IP addresses and ports of the communication servers used in the application are specified. The Start () method establishes the connections and checks their status. If the connections are successfully established, the communication status is updated, and the appropriate message is displayed. Otherwise, the Boolean variable error is set to true, and the error status is reported to the MR interface. If a connection fails during the process, the application triggers an error and stops the communication operations associated with the failed connection until it is reestablished. The automatic, sequential, and safety-related functions of the industrial line operate independently of the MR application; these functions are performed and controlled by the PLC and the associated industrial machinery without relying on the MR application.

### 4.3. Collection of Data and Information

The developed system accepts state and operational data from the industrial assembly line, along with the data received from the network analyzer and the sensors placed on the assembly line. Data is collected periodically based on application logic, which requests the necessary data from the devices and updates the respective values in the MR application. Collected data are used to monitor electrical parameters and the status of the sensors and track changes in the respective parameters and statuses when the system is operating. The period between two consecutive data acquisition cycles, set in the software configuration, equals 10 ms. This period is equal to the nominal value of 100 Hz, but it is not the experimentally validated continuous data acquisition rate. It is the nominal time after which data acquisition requests are launched by the MR application. Actual data acquisition process characteristics are determined experimentally and described in the measurements section. The data acquisition period indicates the scheduling of the data requests during the communication processes, and it is different from the rate of refreshing of the graphical components of the MR interface. The data are presented in the form of variables suitable for presenting those data. The electrical parameters are presented in the form of integer variables, because of their representation in the network analyzer, while the sensors status is represented in the form of Boolean variables. The workflow is presented in Algorithm 2.
**Algorithm 2:** Reading Industrial Parameters**1. Given:** process and electrical parameters **2. Initialize:** parameter values **3. function** Start **4.**   open OPC UA connection **5.**   open Modbus TCP connection **6.**   start Read_Data loop **7. end function** **8. function** Read_Data **9.      while** running **do** **10.**         read sensors parameters from OPC UA server **11.**         read electrical parameters from Modbus TCP server **12.     end while** **13. end function** **14. function Update** **15.**     display process parameters in UI **16.**     display electrical parameters in UI **17. end function** **18. function** Close_connection **19.**      running ← false **20.**      close OPC UA and Modbus TCP connection **21. end function** **22. return** real-time industrial data

To acquire the data, the structure of the script contains several functions that have been assigned a definite task during communication, data acquisition, and value updating processes. First, there are two communication protocols used to establish connections with the industrial line: OPC UA and Modbus TCP. Next, the data acquisition function retrieves data from two data sources and transfers it to the data processing components of the application. The data acquisition process is controlled by the Boolean variable “running,” allowing the process to be started and stopped. Communication with industrial devices takes place using a data acquisition interval of 10 ms configured in the application. This interval corresponds to a nominal frequency of 100 Hz but does not represent an experimentally verified continuous acquisition rate. The actual acquisition performance may depend on the response time of the equipment, communication latency, and application execution time. However, interface elements are updated through the Update() function, which is related to the Unity application update process and the 60 Hz refresh rate of the HoloLens 2 display.

Data acquisition and interface update processes are different from each other. The data acquisition process is configured with a 10 ms interval, while the interface update process depends on the application update period and the refresh rate of the display device. When the process ends, or the “running” variable becomes false, data acquisition stops and the connections to the hardware are terminated. In case of a communication failure, the application stops acquiring new data and alerts the user about the current connection status. This ensures that only valid and available data are used for process monitoring and supervisory commands. Data obtained using the OPC UA and Modbus TCP protocols is used for the monitoring of process variables and the production line status. The data obtained is stored in the database using a separate data processing workflow implemented with Node-RED. The data is processed and distributed by Node-RED nodes according to the structure of the database. The process of storage and treatment of the data in this case occurs independently of the MR–PLC communication used for monitoring of the processes and supervision commands. In the process of experimental data processing, consecutive data are smoothed if needed using the simple moving average (SMA(50)), where 50 data points are considered, to reduce the influence of the short-term fluctuations on the data. The aim of this process is to smooth the data and enhance the trend in the figure without changing the original data stored in the database. The use of 50 data points in this process reduces the local variations and helps to analyze parameter trends in the figures.

### 4.4. Data Processing and Control of the Industrial Assembly Process

The parameters of the industrial assembly line selected for analysis by the mixed-reality system are the electrical parameters measured by the power analyzer. These parameters characterize the industrial process under varying operating conditions of the assembly line. The statistical analysis of the collected data is performed using descriptive statistical methods, including the minimum, maximum, mean, median, standard deviation, and coefficient of variation. These measures characterize the magnitude and variability of the electrical parameters and allow their behavior to be compared across different operating conditions of the assembly line. Linear regression is additionally applied to describe the temporal evolution of the electrical parameters. Here, time or the sample index is the independent variable, whereas the dependent variable is the analyzed electrical parameter, such as voltage, current, power, or power factor. This type of analysis is applied descriptively to determine the trend direction and trend rate by analyzing the slope of the regression line, but it is not applied for state classification or detection of defects. The operational condition of the manufacturing line is determined from the operational state information provided by the automation system, while the monitored electrical parameters are analyzed separately to characterize their behavior and variability under different operating states. To reduce short-term variability and highlight the overall trend of the data sequence, a simple moving average with a 50-sample window (SMA(50)) is applied when necessary. The simple moving average is calculated from successive values of the studied parameters and is used to smooth local variations and facilitate the interpretation of parameter behavior. The next part describes the statistical methods applied to the characterization of electrical parameters. Calculation procedures and meanings of the statistics are described without considering any basic mathematics formulas; the focus is made on the practical application of the calculations.

Standard deviation

(1) $$\sigma =\sqrt{\frac{\sum_{i=1}^{n}{\left(x_{i}-\mu \right)}^{2}}{n}}$$

- *x_i_*—individual values;
- µ—average of values;
- *n*—total number of values.

**Coefficient of variation**

(2) $$\mathrm{CV}=\frac{\sigma }{\mu }\cdot 100$$

- *σ*—standard deviation;
- *μ*—arithmetic mean.

**Median**

For an odd number of values:

(3) $$\mathrm{Me}=x\frac{n+1}{2}$$

For an even number of values:

(4) $$\mathrm{Me}=\frac{x\frac{n}{2}+x\frac{n}{2}+1}{2}$$

- Me—median;
- *x_i_*—values sorted in ascending order;
- *n*—total number of values.

**Linear regression**

*y* = *a* + *bx* (5)

- y—estimated value;
- x—independent variable;
- a—the value of y for x = 0;
- b—the slope of the regression line.

**The coefficients are calculated using the following formulas:**

(6) $$b=\frac{n\sum x_{i}y_{i}-\sum x_{i}\sum y_{i}}{n\sum x_{i}^{2}-{(\sum x_{i})}^{2}}$$

(7) $$a=\bar{y}-b\bar{x}$$

- **n**—data count;
- $\bar{x}$—average of the values x;
- $\bar{y}$—average of the values y.

The mixed reality system enables the operator to select the operating mode of the industrial assembly process, configure the parts, and issue supervisory commands to start, stop, reset, and reconfigure the process. These actions are carried out using the supervisory control panel of the MR application. The commands generated by the MR program are high-level managerial commands, while the sequencing of the process itself is controlled by the PLC. Before sending a command, the MR application validates the communication link status and the conditions required for command execution. The corresponding command is then sent to the PLC using the OPC UA protocol. The PLC receives command and acts accordingly based on the control logic implemented for the assembly line. Hence, the MR application provides supervisory control and monitoring capabilities without interfering with the control logic, interlocks, or safety functions implemented in the PLC. The industrial assembly line supports multiple part configurations. There are 12 different positions for rectangular parts defined by the configuration matrix. The data related to the chosen configuration are associated with the RFID tag attached to the base of the blue parts and are transferred to the PLC as high-level configuration data. The application verifies whether sufficient adjacent positions are available in the matrix for placing the rectangular parts. A rectangular part may be placed only if two adjacent positions are available in either the horizontal or vertical direction. Thus, a position can be selected only when an adjacent available position allows the part to occupy two positions. Once the configuration is sent, the PLC controls the corresponding process steps and sends information to each machine. In such an architecture, the MR system would define the chosen configuration and send out supervision signals whereas the PLC would be responsible for carrying out the process steps according to the operating conditions defined by the control logic. The final configuration may consist of a maximum of four rectangular subcomponents, depending on the number of available positions and the restrictions placed on the configuration matrix occupancy. The configuration algorithm analyzes the available positions in sequence and enables the selection of a configuration only if the occupancy requirements are satisfied. The logic behind this process is explained through the pseudocode presented in Algorithm 3.
**Algorithm 3:** Parts configuration**1. Given:** occupancy matrix M [3,3] **2. Initialize:** M [i,j] = 0 **3.** k = 1 **4. while** k ≤ Np **do** **5.**  o ← O(k), o ∈ {H,V} **6.**  (i,j) ← P(k) **7.  if** V(i,j,o) = 1 **then** **8.       if** o = H **then** **9.**           M(i,j) ← k **10.**        M(i,j + 1) ← k **11**.     else **12.**        M(i,j) ← k **13.**        M(i + 1,j) ← k **14.     end if** **15.**     k ← k + 1 **16.  else** **17.**     (i,j) ← Next(i,j) **18.  end if** **19. end while** **20. return** M

The above algorithm uses a state matrix and a set of variables to arrange the pieces within the given rectangular shape. The available positioning area is shown by a 3 × 3 square matrix (M). During initialization, all the elements in matrix (M) are initialized to zero, meaning that the respective positions are unoccupied. The number of pieces to arrange is denoted by the variable (N_p), with a maximum of 4 pieces. Two consecutive positions in matrix (M) are selected for each piece based on the dimensions and orientation of the rectangular piece. The piece can be arranged either vertically or horizontally, and the availability of the corresponding positions is checked before the configuration is assigned. In a vertical arrangement, the selected position and the adjacent position in the same column are selected for checking their availability. If the respective positions are available, the corresponding elements in the matrix (M) are assigned the value of the piece identifier, with values ranging between 1 and 4. Similarly, for a horizontal arrangement, the same criterion is applied, but using two adjacent locations in the same row.

Variables (i) and (j) are the row and column indices of the current location in the matrix, respectively. To identify the next candidate location, the Next(i,j) procedure is used to update the index pair according to the relationship defined in the algorithm. After evaluating all the candidate locations and verifying the configuration, matrix (M) is obtained as the resultant occupancy status of the configuration zone. Matrix (M) is then sent to the PLC through OPC UA communication. The PLC then utilizes this configuration information as an input to the predefined process-control logic for process step coordination and execution, including control of the robotic arm through the appropriate control logic. Thus, the MR application is used for operator interfacing, configuration selection, position availability verification, and configuration transmission to the PLC, while the PLC is responsible for the process-control logic and execution.

The system operation can be done in two ways; these include automatic and manual operating modes. For automatic mode, the application transmits the predefined configuration to the PLC, where the process sequence is executed according to the control logic contained in the PLC. However, in manual mode, the operator selects the configuration and orientation of the parts. The MR application then checks the availability of the selected positions and, when a valid configuration is obtained, transmits the configuration to the PLC. In both operating modes, the mixed reality system acts as an interface for process monitoring and supervisory control by transmitting configuration information and supervisory commands, while the sequential control logic remains implemented and executed in the PLC.

## 5. Results

Experimentation for the validation of the system was carried out in about 40 experiments divided into four experimental scenarios, with 10 repetitions of each scenario: OPC UA Read over Ethernet, OPC UA Write over Ethernet, Modbus TCP over Ethernet, and OPC UA Read over wireless communication. Each experiment was conducted in the seven operating modes of the industrial line. For each operating mode, 1500 values of electrical parameters were measured, resulting in 10,500 measurements for each experiment and 105,000 measurements for each experimental scenario. Data acquisition for each operating mode took about 150 s, resulting in about 1050 s for one full experiment consisting of all seven operating modes. The evaluation of the communication performance was performed by capturing and analyzing network packets using Wireshark. The Round-Trip Time (RTT) of the communication packets in each communication case was calculated as the time elapsed between sending a request and receiving the corresponding response. Further statistical analysis of the obtained RTT data was performed for each test case. Instead of considering only the average value, communication latency was characterized using the median, standard deviation, 95th percentile (P95), and 99th percentile (P99). The corresponding statistical results are provided in Table 3.

The Ethernet channel with a data rate of 1 Gbps was used for the wired communications, whereas the ZTE MF289F LTE Cat. 20 router was used to conduct wireless communication experiments. The experimental system included a Siemens S7-1500 PLC and a Microsoft HoloLens 2 mixed-reality device. During the experiments, the mixed-reality system established direct communication with the main PLC of the industrial assembly line without any additional intermediate devices for data transmission between the MR application and the PLC. Meanwhile, the PLC remained responsible for communication with the devices of the industrial line. The wireless communication tests were conducted under the conditions of the connection provided by the LTE router used during the experiments. Thus, the observed RTT fluctuations reflect the conditions of the experimental wireless connection and should not be attributed exclusively to network traffic or to wireless communication in general.

**Figure 9:**
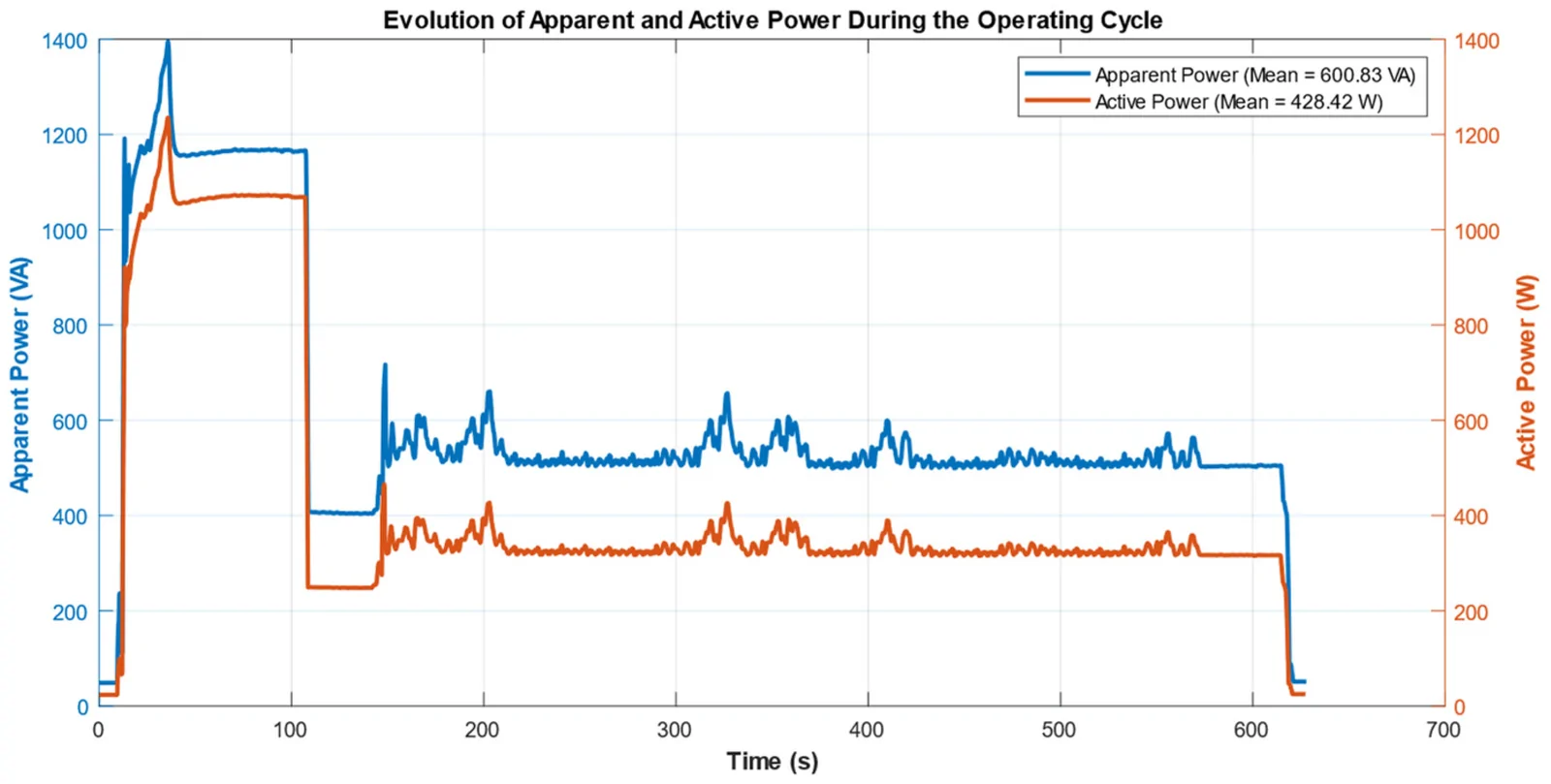
Evolution of apparent and active power during the operating cycle.

Figure 9 shows the changes in apparent power and active power over one operating cycle of the industrial assembly line, which corresponds to one of the operating modes studied. At the beginning of the cycle, both indicators increased due to the start-up of the equipment. As the cycle progresses, the indicators stabilize at relatively constant levels within the operating mode, with fluctuations associated with transitions between different stages of the assembly process. At the end of the cycle, both apparent power and active power decrease to near-zero levels. The results obtained indicate a correspondence between the measured electrical parameters and the different phases of operation of the assembly line. The observed variations in equipment operation are reflected in corresponding changes in the measured electrical parameters. The purpose of the analysis was not to automatically classify the operating state, but rather to examine the electrical behavior of the assembly line under different operating modes. To reduce measurement fluctuations and highlight the general trend of the parameters, the data shown in the figure was processed using the Simple Moving Average (SMA) method.

**Figure 10:**
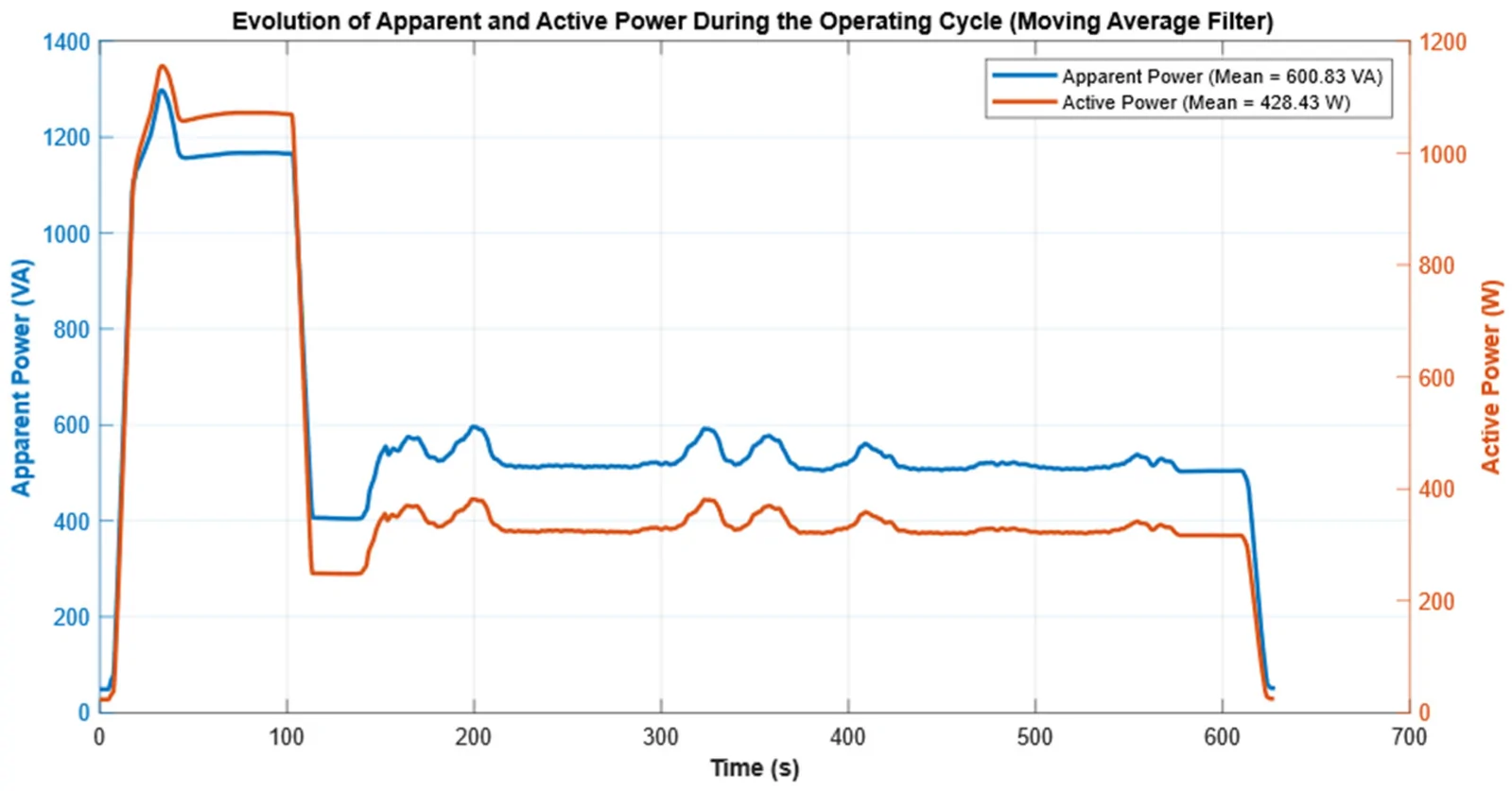
Evolution of Apparent and Active Power during the Operating Cycle Using SMA.

The following method is used for processing the two electrical parameters presented in Figure 10—the Simple Moving Average (SMA) technique with a window of 50 values, referred to as SMA(50). It will minimize small fluctuations in the measured data and help us to see more clearly the overall trends in the parameters and the changes in them from one operating mode to another.

**Figure 11:**
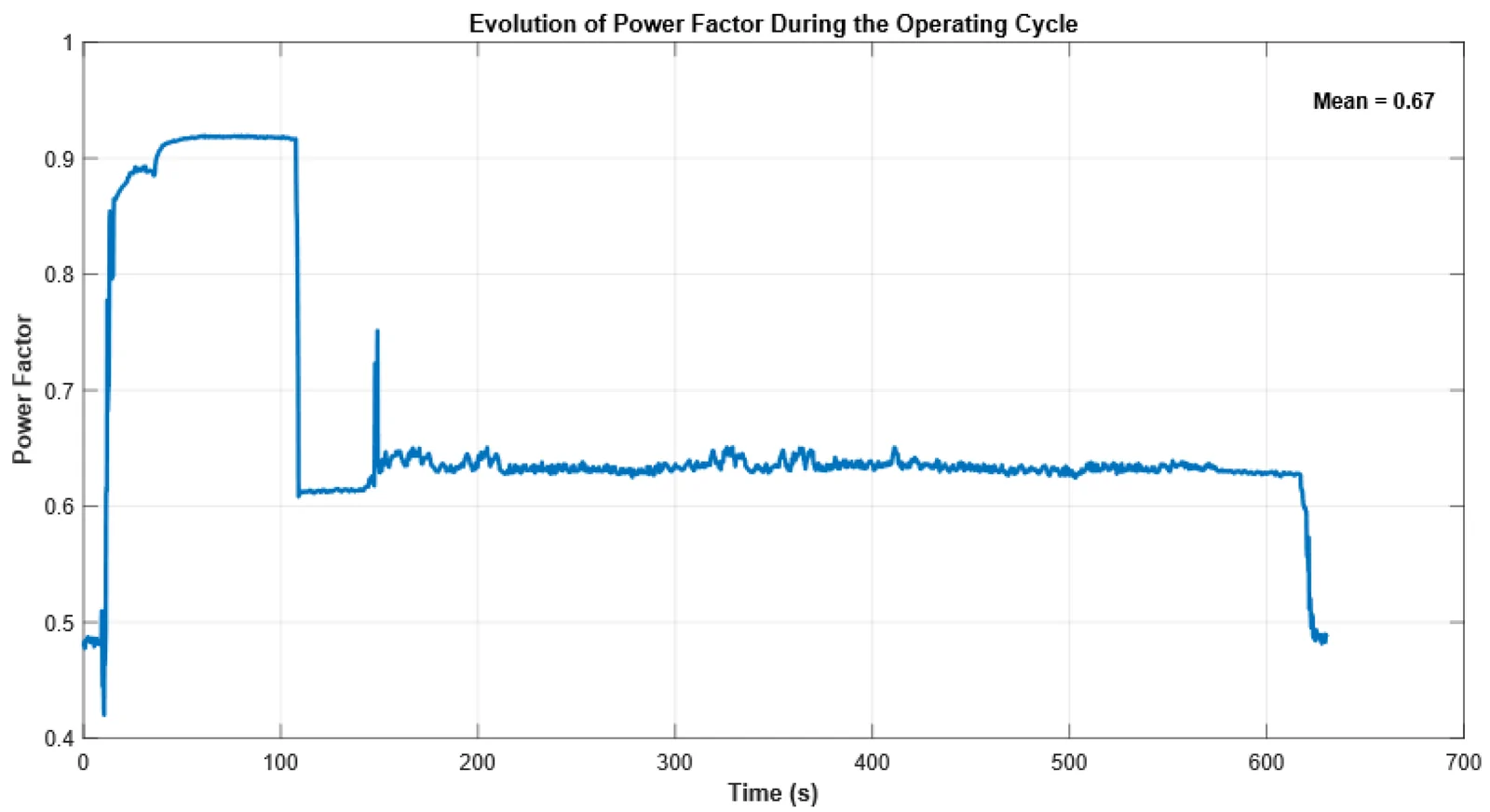
Evolution of Power Factor During the Operating Cycle.

The power factor curve for an industrial line operating cycle is illustrated in Figure 11. In the first stage of the operating cycle, the power factor rises from 0.48 to 0.92, which relates to the startup period and the stable functioning of the equipment. Further, the power factor declines to 0.61 and fluctuates slightly around this value due to changes in the operating conditions of the equipment. In the last stages of the operating cycle, the power factor declines to 0.49, which corresponds to the equipment shutdown period. Thus, the change in the power factor illustrates different variations that are characteristic of different operating phases of the line.

**Figure 12:**
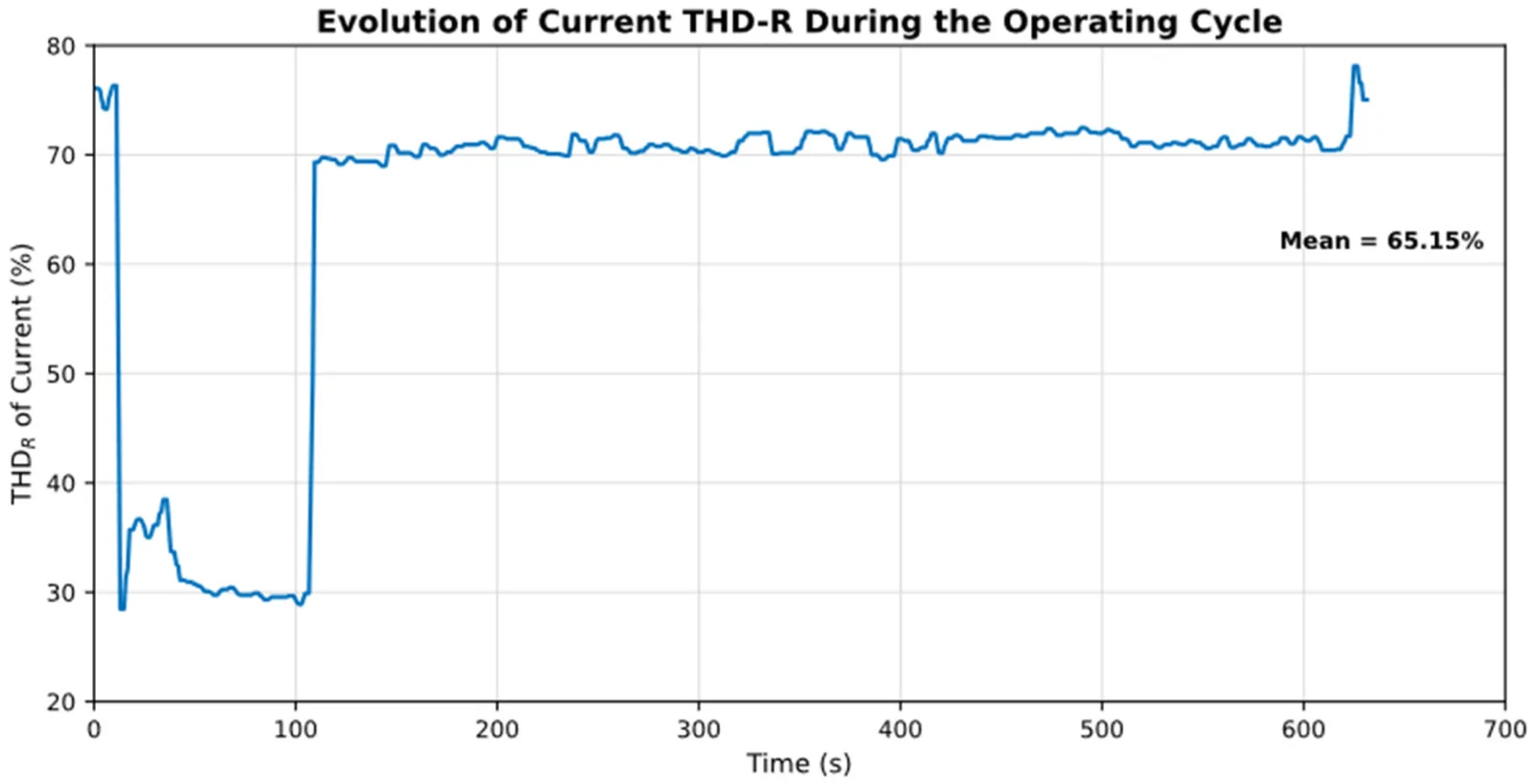
The evolution of total harmonic distortion of the current (THD-R) during the operating cycle.

As is apparent from Figure 12, the THD-R values for the current show more pronounced variations during the start-up period, then they increase and become relatively stable during operation. At the end of the cycle, variations in the THD-R values are again detected, specifically now when the line changes its operating mode and enters the shutdown phase. During steady-state operation, the THD-R values vary to a lesser extent than during the periods of transition between operating modes.

**Figure 13:**
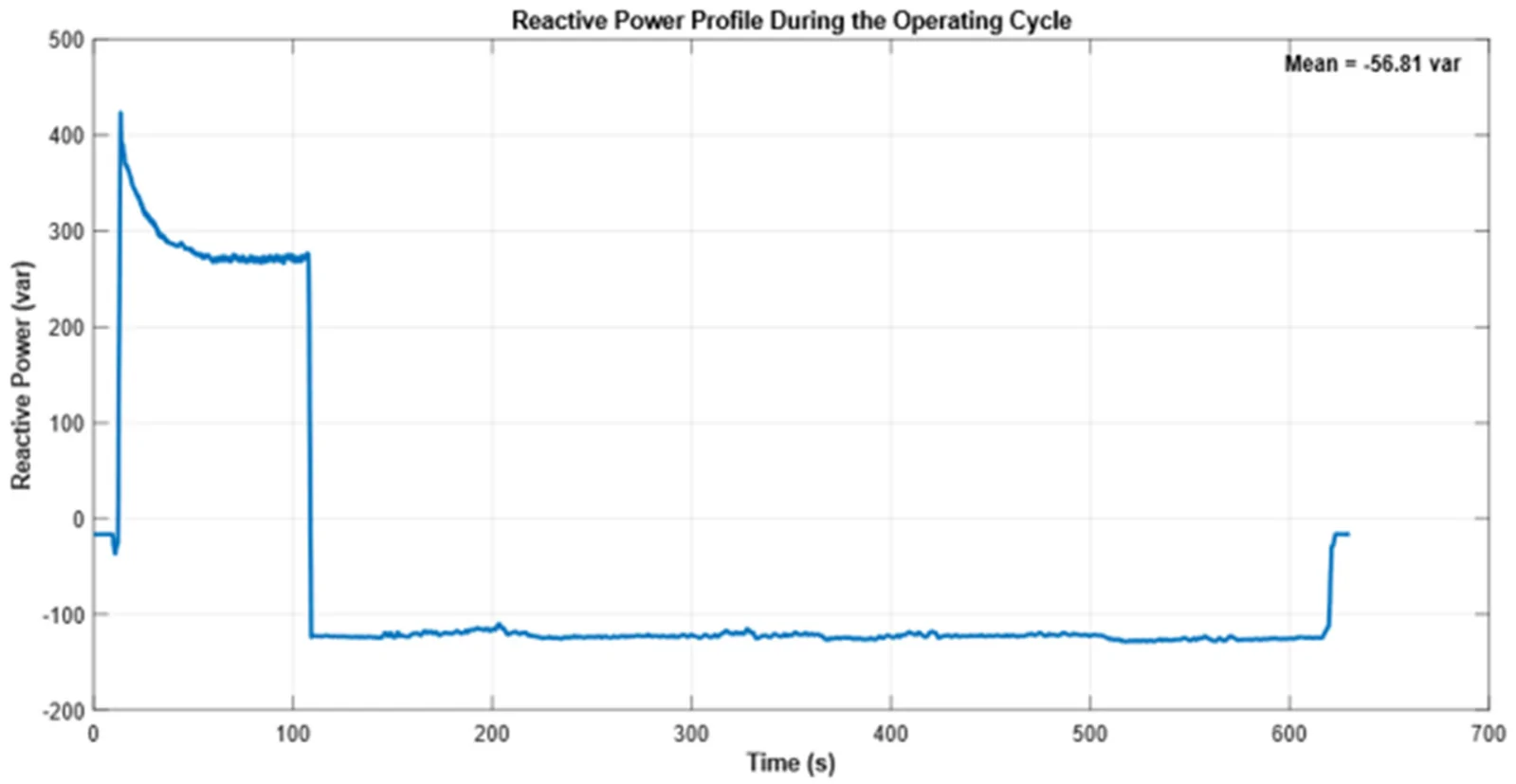
Variation in Reactive Power During the Operating Cycle of the Industrial Assembly Line.

**Figure 14:**
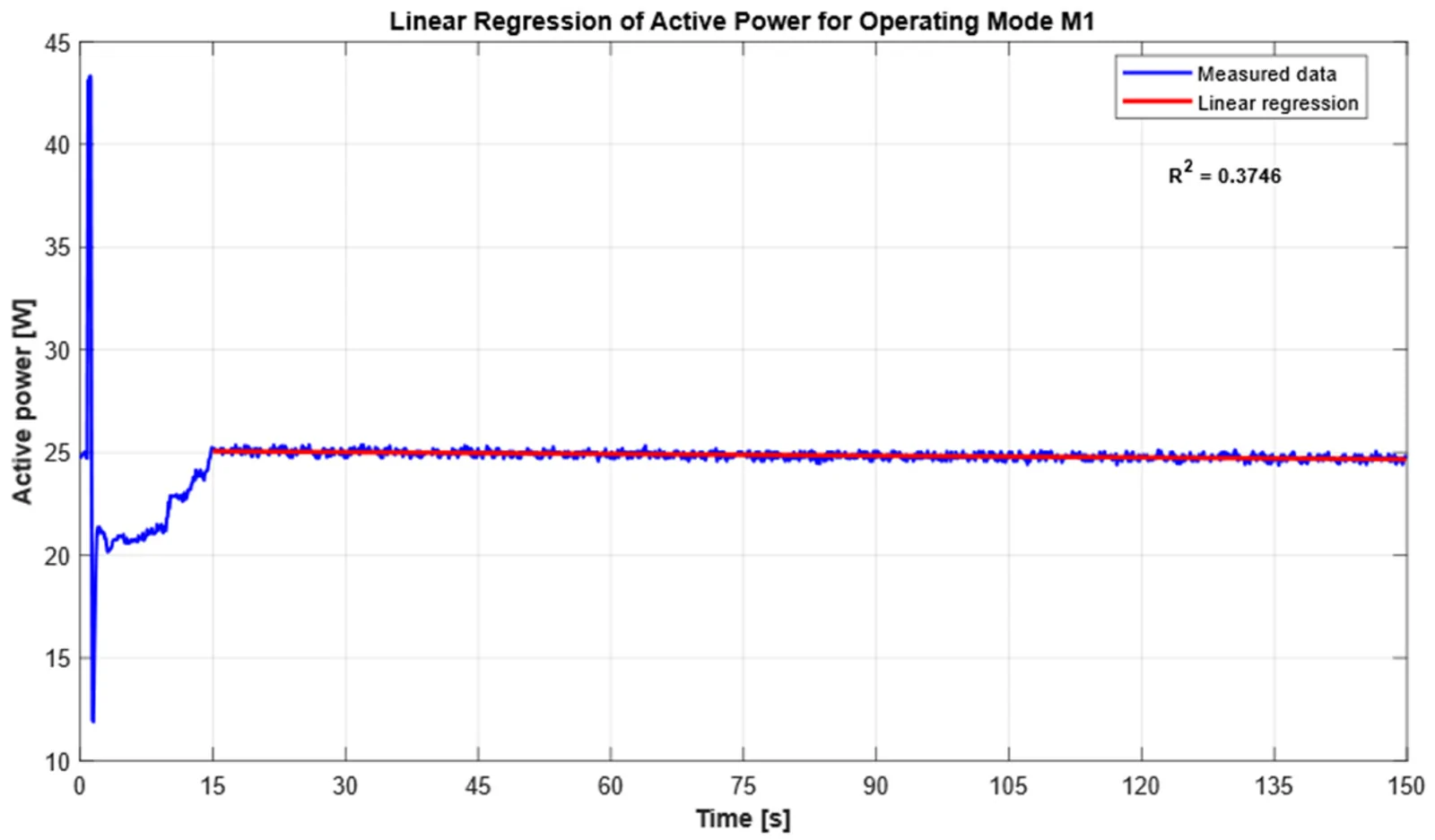
Linear regression for operating mode M1.

The evolution of reactive power during the operating cycle is represented in Figure 13. It is possible to note that during the equipment startup stage, there are high values of parameters and rapid changes; further, its value decreases due to approaching a more stable operating regime. In steady-state operation, reactive power has constant values; nevertheless, variations occur at the end of the cycle when the equipment switches from the operating mode to the shutdown mode. The mean value of reactive power for the analyzed cycle is equal to −56.81 var. To describe the dynamics of the electrical parameters during the operation of the industrial line in various modes, the method of linear regression is employed. The goal of the analysis is to reveal the temporal trends in the parameters within each operating mode and to find out if there is an approximately linear dependence of the parameters within the interval corresponding to the mode considered. Several operating modes of the industrial line were revealed, and the method of linear regression was applied separately for each mode. As such, linear regression is only employed as a means of describing the tendencies in the data obtained from the experiment and the changes in the electrical parameters for each mode of operation. It is not used for the automatic classification of the operating state or for defect identification. Figure 14 shows the evolution of active power along with the linear regression line for the first mode of operation of the industrial assembly line.

It can clearly be observed from Figure 14 that the coefficient of determination is R^2^ = 0.3746. In other words, the variation of active power cannot be considered to follow a significant linear trend. After a certain transitional value, there is very little variation in the active power, which remains steady at about 25 W.

**Figure 15:**
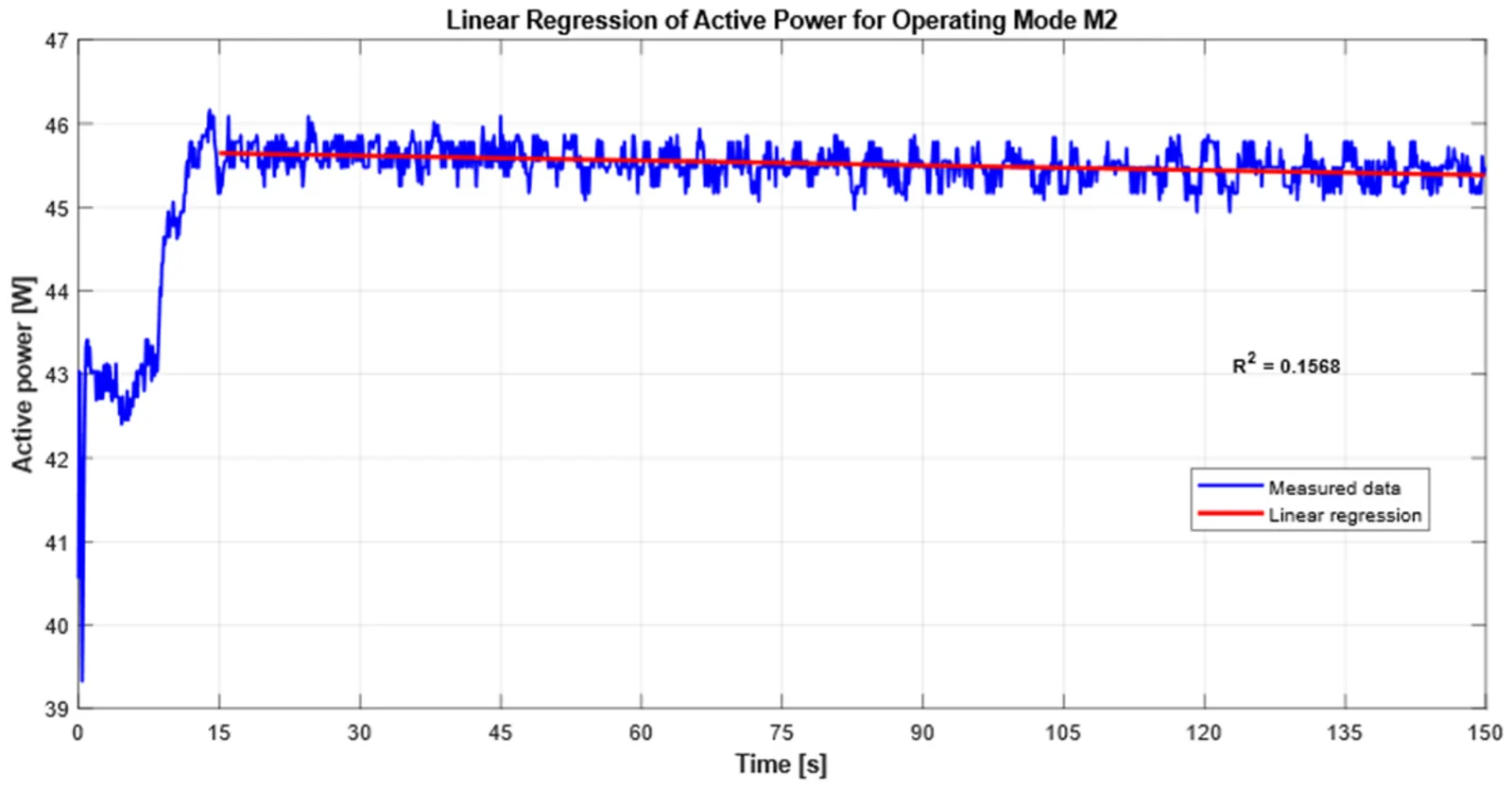
Linear regression for operating mode M2.

Active power variation for the second regime of the industrial assembly line operation is presented in Figure 15. The coefficient of determination is R^2^ = 0.1568. It means that there is a low correlation between time and active power. Active power varies slightly around 45 W after the transition stage. Thus, linear regression analysis mainly performs a descriptive role in this situation.

**Figure 16:**
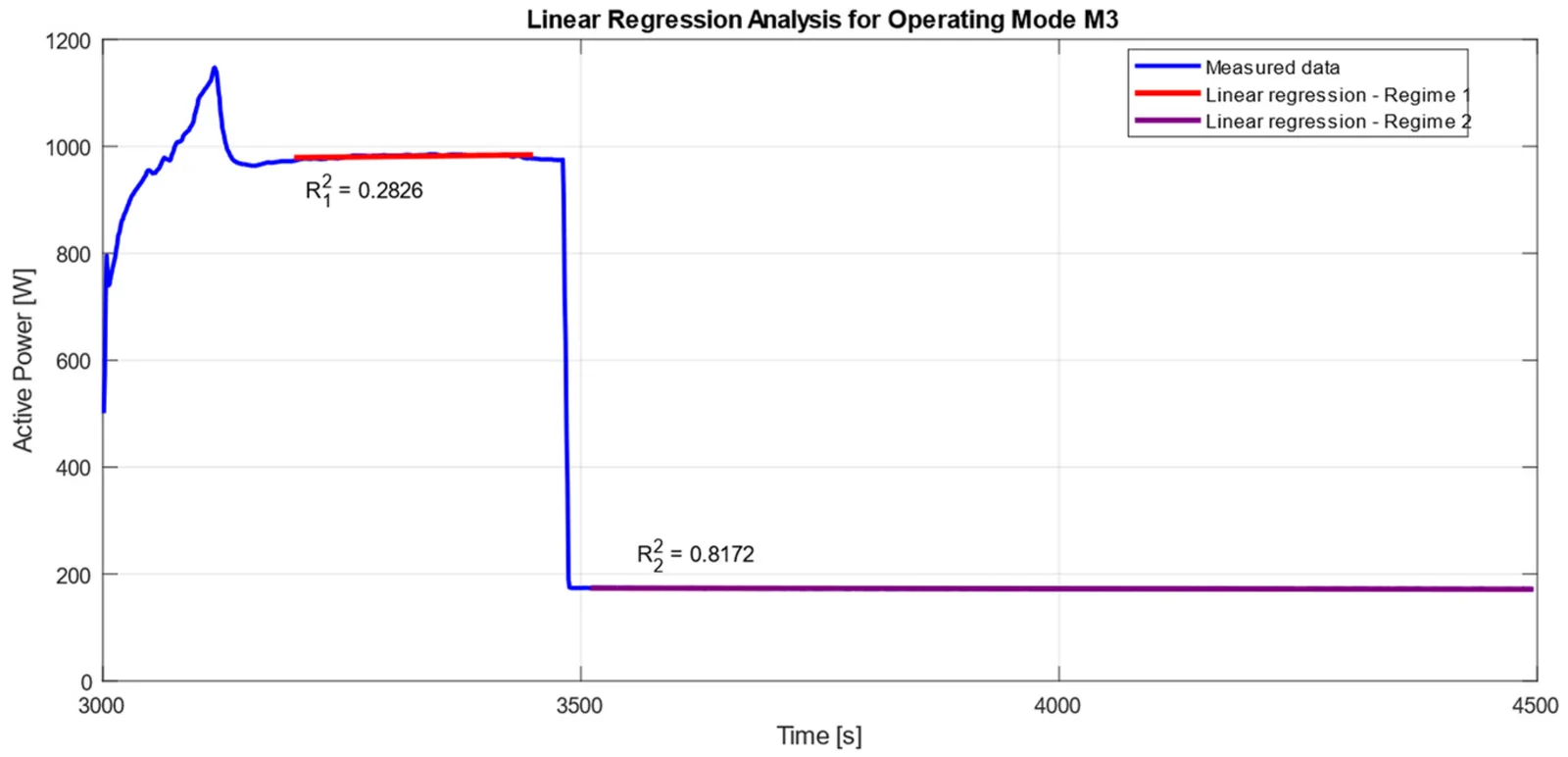
Linear regression for operating mode M3.

The two regions used to describe the third mode of operation of the assembly line correspond to different trends in the active power. In this case, the two regions have been considered to have different values of active power. For this reason, the linear regression has been performed on the two regions separately, thus not using the same straight line for the whole period (Figure 16). In the first region, the coefficient of determination is R^2^ = 0.2826. In the second region, the coefficient of determination is R^2^ = 0.8172.

**Figure 17:**
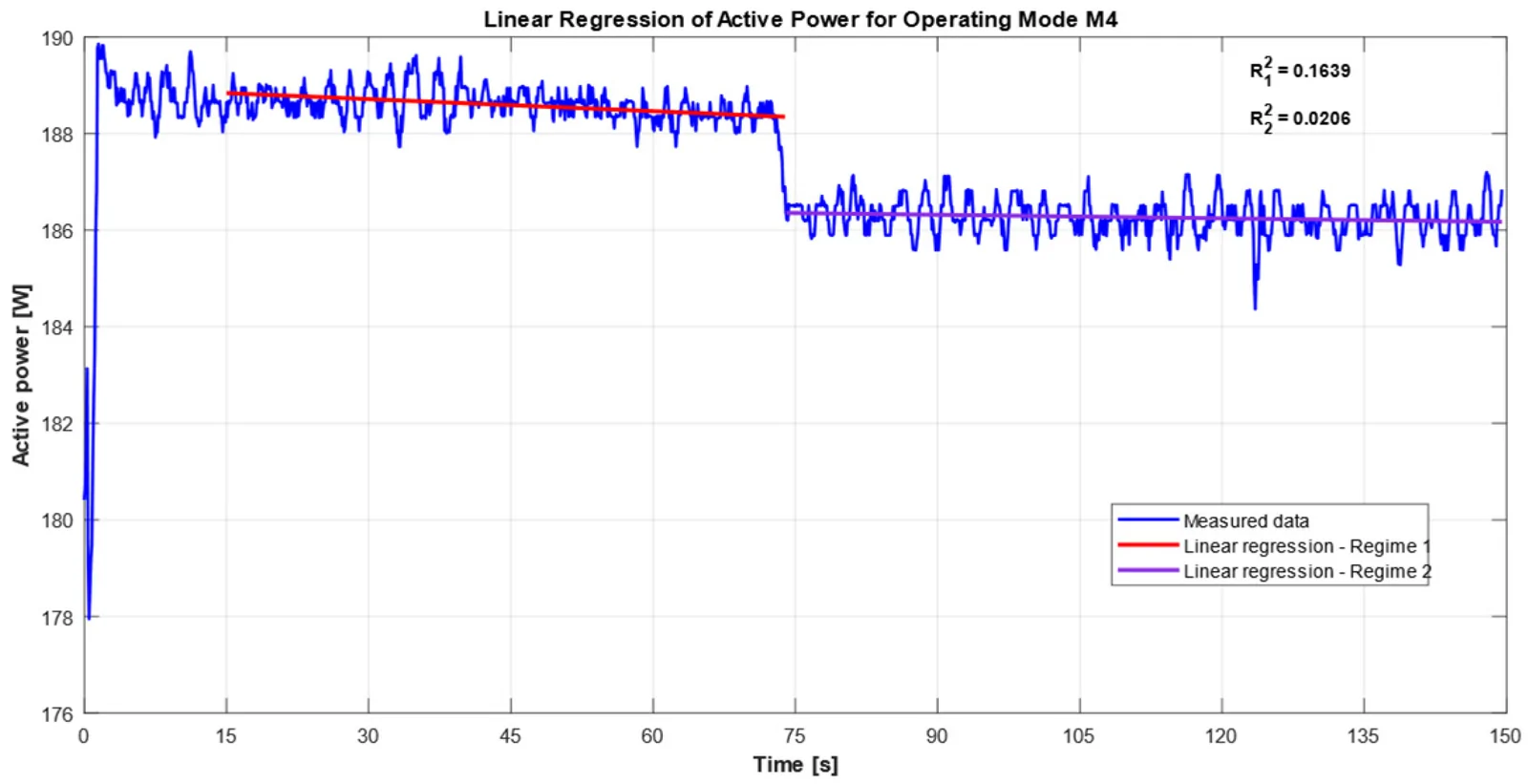
Linear regression for operating mode M4.

Regression analysis for operation M4 is presented in Figure 17. As the level of active power changes at approximately 73 s, regression analysis has been carried out for both operation modes. For the first operation mode, the coefficient of determination is R_1_^2^ = 0.1639, whereas for the second operation mode, it is R_2_^2^ = 0.0206. Considering the low value of the coefficients of determination, changes in active power cannot be explained by a linear trend.

**Figure 18:**
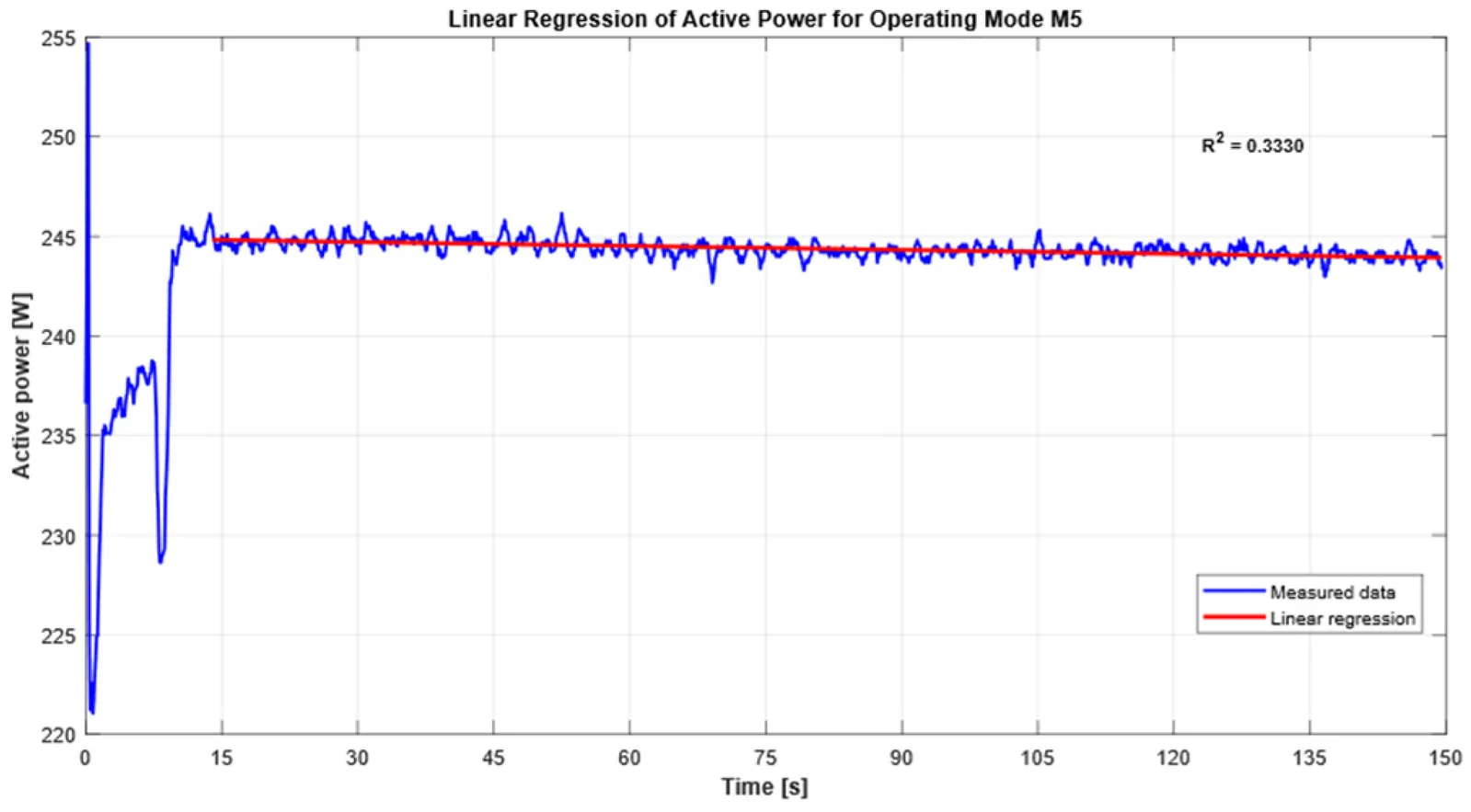
Linear regression for operating mode M5.

The graph of the active power in the operating mode M5 is represented in Figure 18. The coefficient of determination is R^2^ = 0.3330, which shows that the relation between time and active power is weak to moderate over the range studied. After the period of adaptation, the active power stays at a level relatively close to 244–245 W.

**Figure 19:**
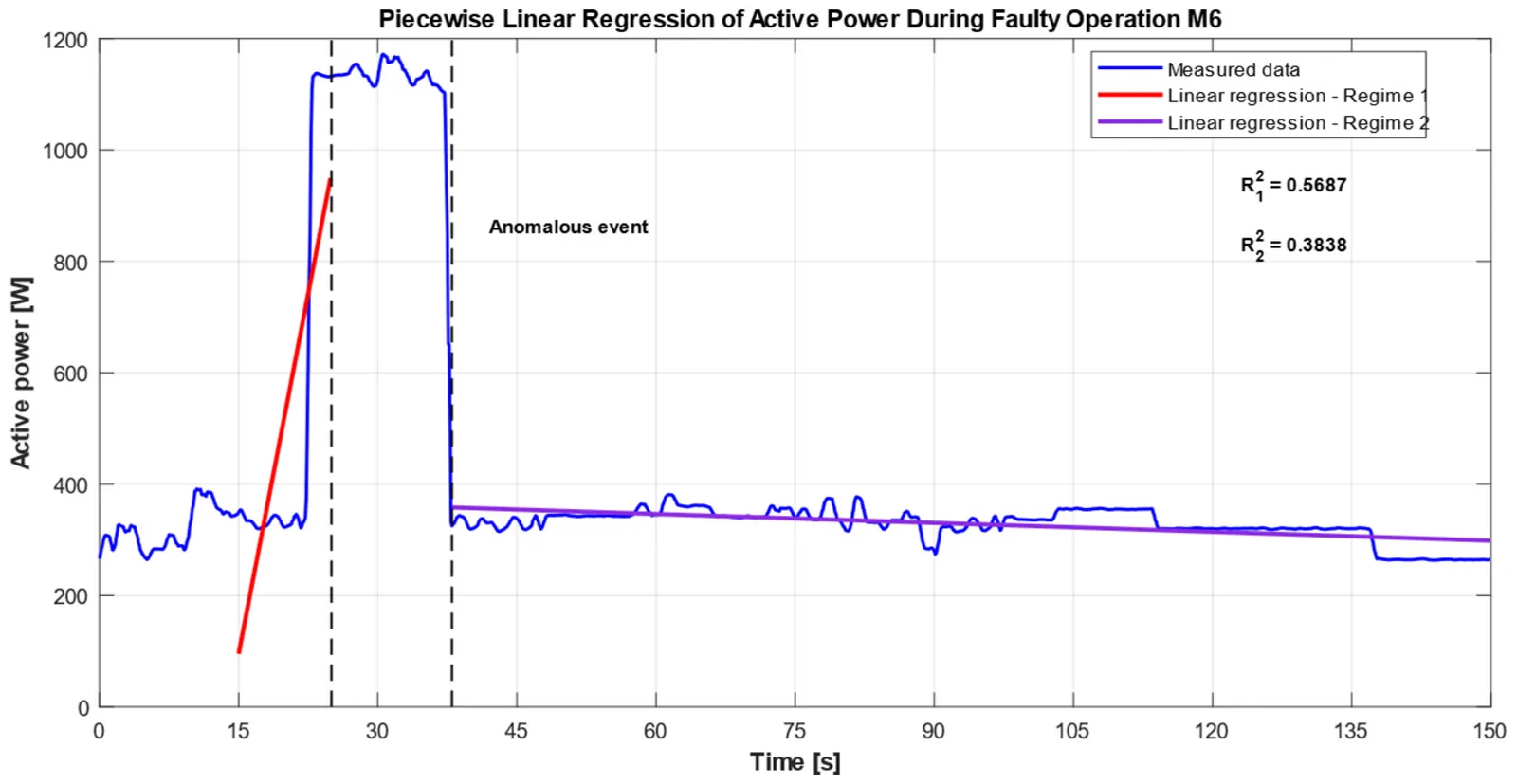
Linear Regression for Operating Mode M6.

Figure 19 presents the M6 operational regime of the assembly line in the industry, which corresponds to the anomaly regime. Under this regime of operation, there is evident non-stationary activity in the active power in terms of fluctuation measurements and a power surge during the time interval from 25 to 38 s. As a result of this new operating regime, it was not possible to use a single linear regression model throughout the cycle since the 25–38 s period corresponded to a transient regime related to the anomaly. In contrast, linear regression was separately used for the two normal operating regimes that existed prior to and after the anomaly: 15–25 s and 38–150 s, respectively. Coefficients of determination for the two regimes were obtained as R_1_^2^ = 0.5687 and R_2_^2^ = 0.3838, respectively. Hence, the results of the regression analysis are only to be treated as a description of the trends in active power during the respective operating regimes.

**Figure 20:**
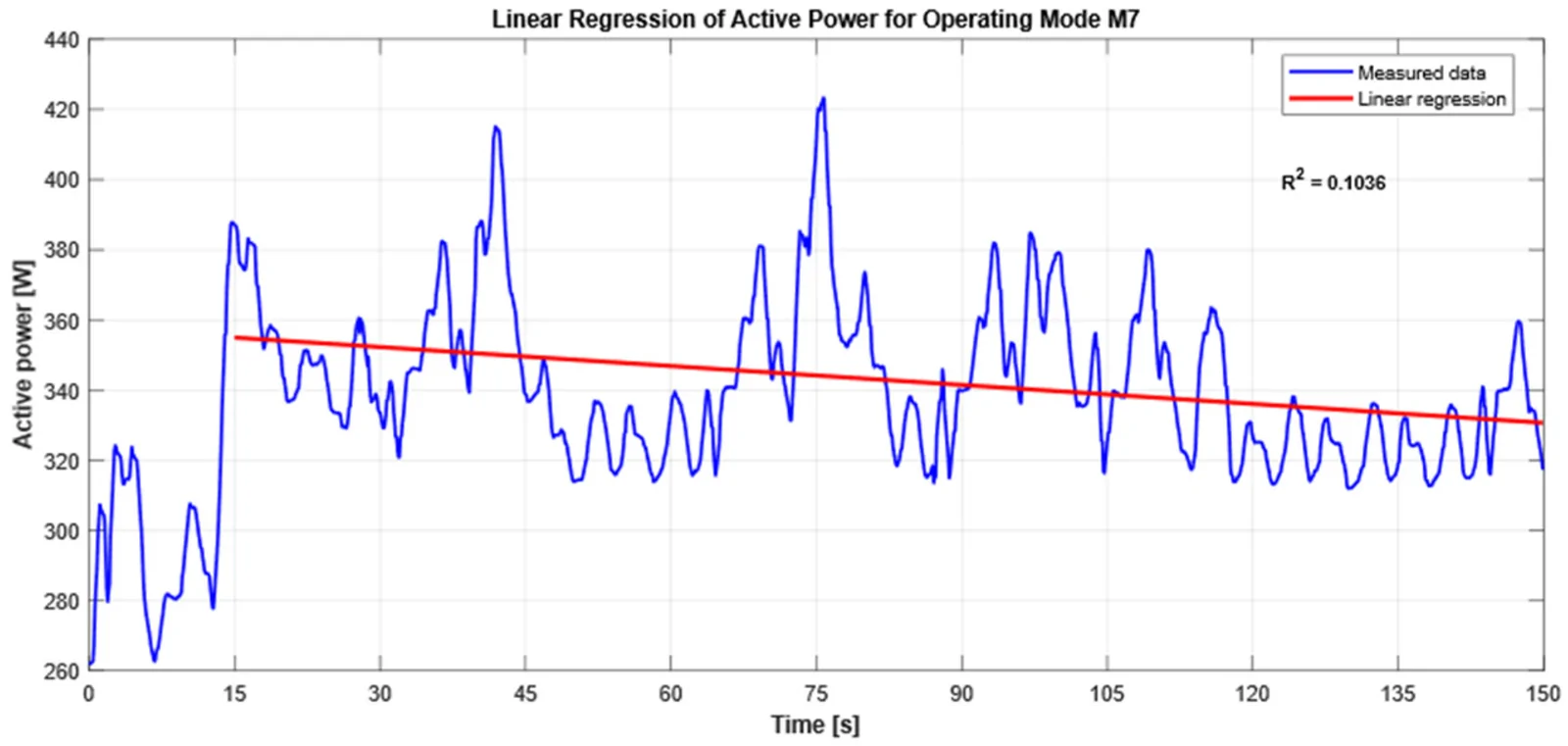
Linear Regression for Operating Mode M7.

Linear regression for the active power of the M7 module, corresponding to the line operating properly, is illustrated in Figure 20. As we can see from the graph, there are substantial local variations in active power taking place at an average value of about 340 W, and there is no trace of any kind of linear dependence during the considered period. The linear regression line slope is negative and rather small, and R^2^ = 0.1036 means that a relatively small part of the total variation of the active power is explained by linear regression. With respect to the line illustrating the normal operating mode, the result emphasizes the stability of the active power range regardless of local variations. To analyze the trends and changes of the data associated with the state of the electrical parameters, several descriptive statistical measures are utilized, including the minimum, maximum, median, standard deviation, and variance. All this data and information help to understand how the operating conditions of the industrial assembly line affect the values of the electrical parameters. To emphasize this effect, these parameters are provided in Table 2.

Descriptive statistics of active power are given in Table 2 for the seven operating modes, that is, M1–M7. The operating modes were determined based on the operating stages and active stations of the industrial assembly line. In M1, one station is operated, while in M2, two stations are operated at the same time. In M3, the third station, including the robotic arm for assembling parts, is operated. In M4, the fourth station is operated, while in M5, the final station of the assembly line is also activated. M6 represents a malfunction stage of the industrial line. In M7, normal operation of the whole assembly line takes place, where the assembled part is transferred to the final station. Traffic on the network was observed using the Wireshark tool to capture communication packets and calculate the communication latency. The protocols considered in the analysis were OPC UA and Modbus TCP, and the types of connections were Ethernet and wireless connections. The communication latency is given in terms of Round-Trip Time (RTT), which is the period between the time when the request is sent and when the response is received. These RTT values were calculated by identifying the corresponding request and response packets and determining the time difference between their timestamps.

**Figure 21:**
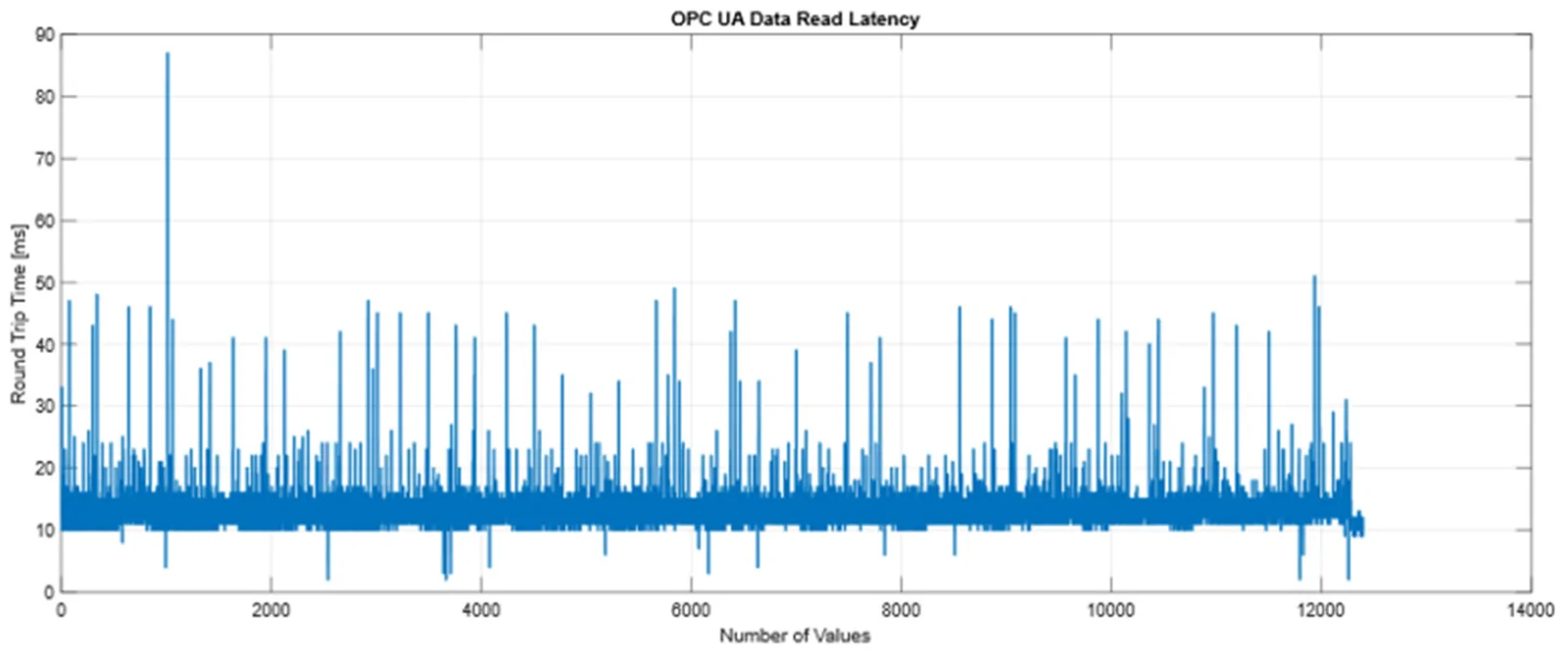
OPC UA data read latency over ethernet.

**Figure 22:**
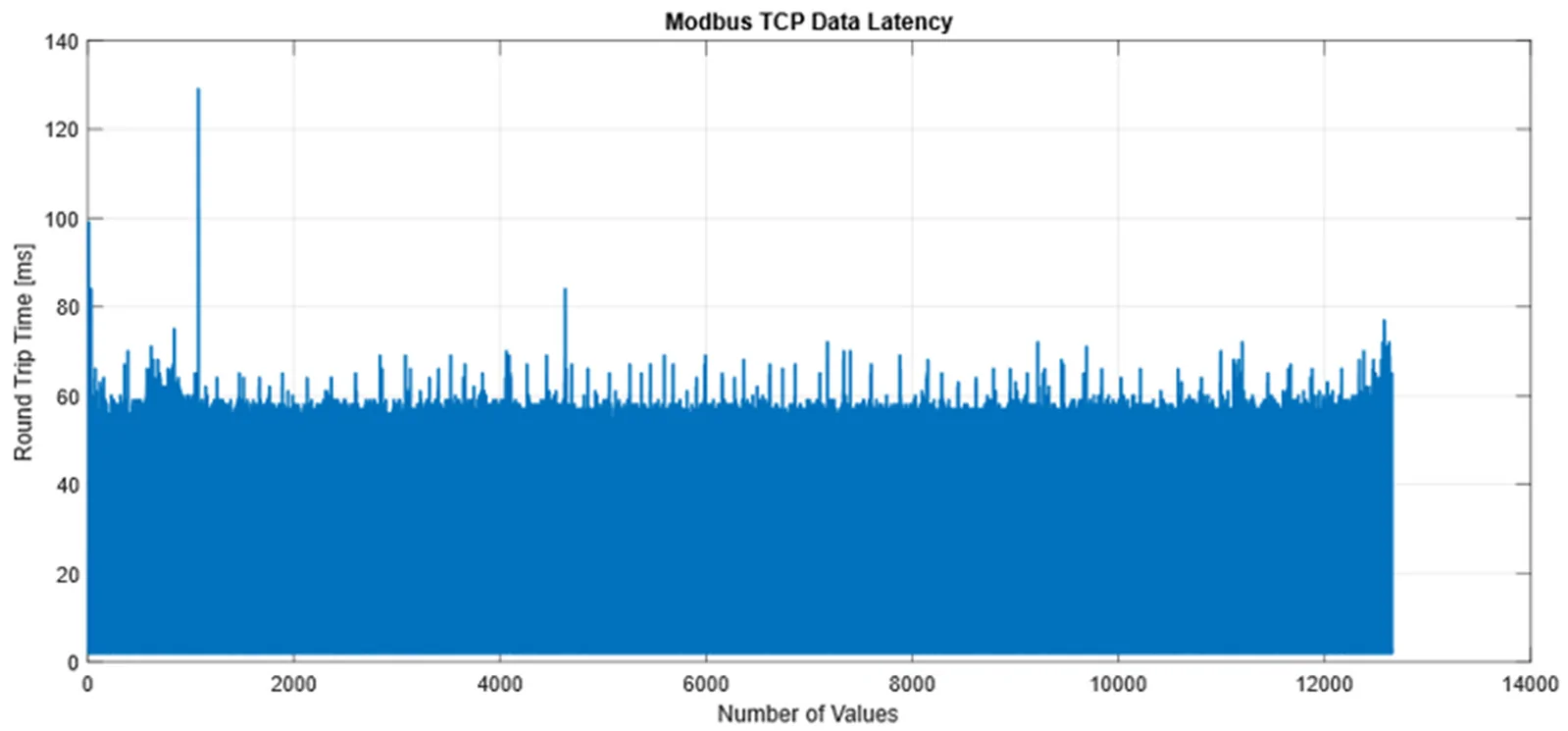
Modbus TCP data read latency over ethernet.

As illustrated in Figure 21, almost all the data is within the range of 10 to 20 ms. There have also been instances where the readings were 50 ms. The maximum value reached was 90 ms. It means that the variation is relatively low, and the connection is stable. For the second test, electrical parameter data was acquired using an Ethernet connection and Modbus TCP. The data trends are illustrated in Figure 22.

**Figure 23:**
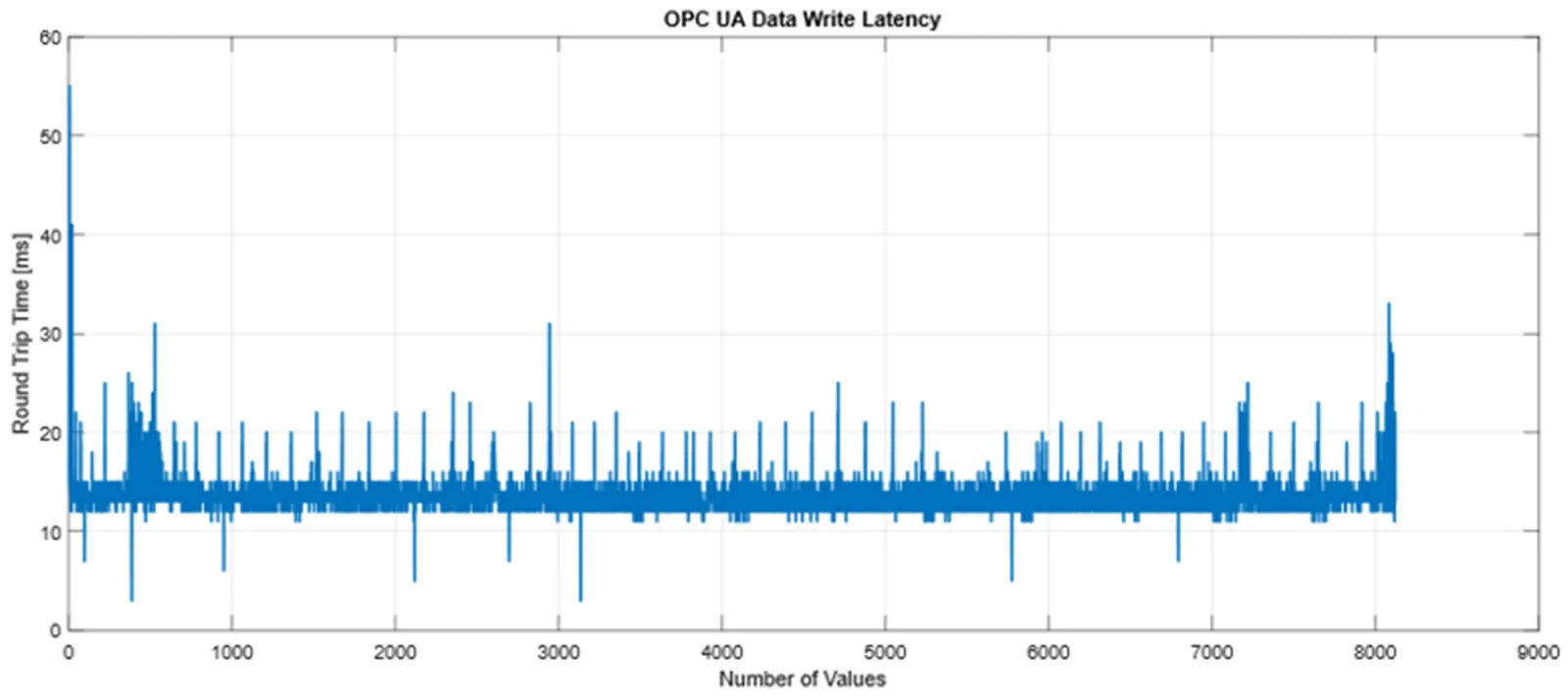
OPC UA data write latency over ethernet.

Latency values for reading data through an Ethernet connection and using the Modbus TCP communication protocol vary from 55 ms to 60 ms. The fluctuations observed here are from 70 ms to 80 ms. The maximum value observed is around 130 ms. In comparison to the OPC UA communication protocol, the Modbus TCP communication protocol leads to the occurrence of higher latency values despite the lower fluctuations observed in the values obtained. In the next test, the OPC UA communication protocol will be used to write data, and the devices will be connected through an Ethernet connection. The graph for this case is presented in Figure 23 below.

**Figure 24:**
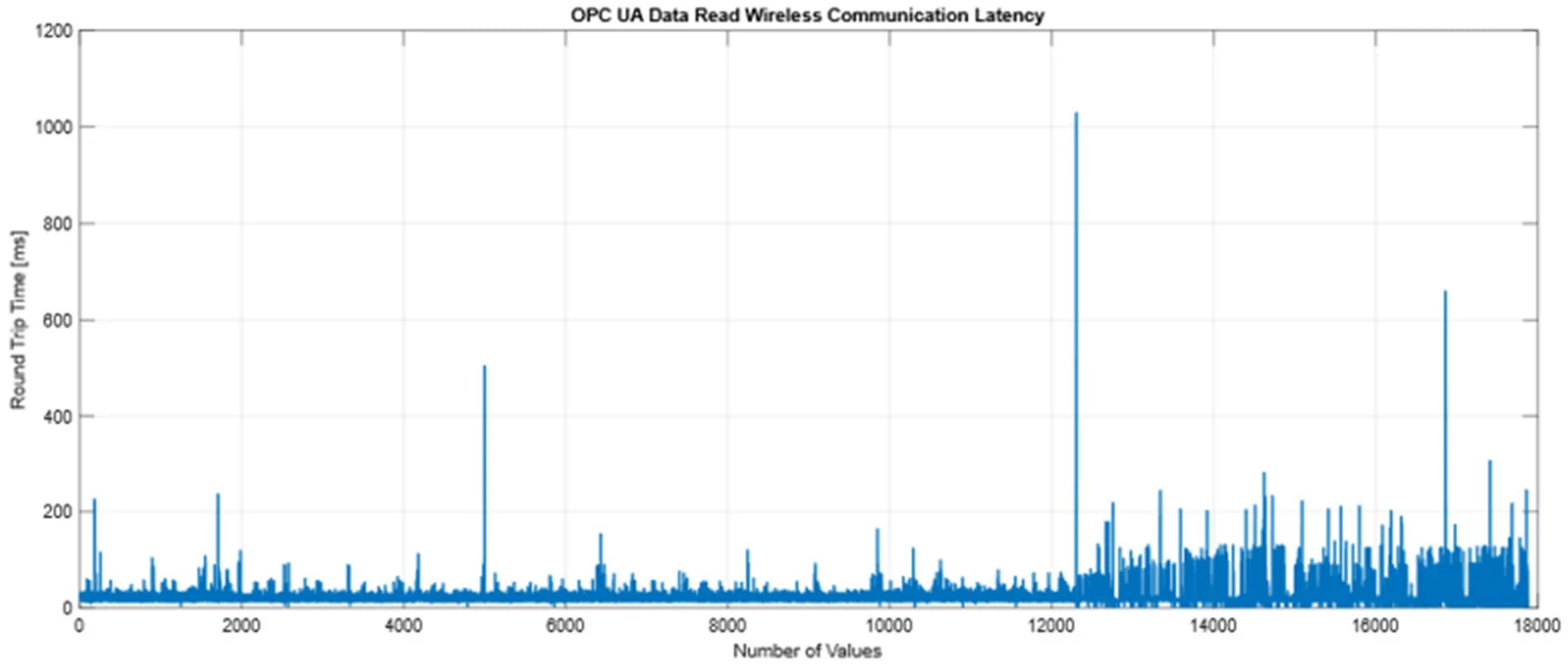
OPC UA Data Read Latency Over Wireless.

As we can see from the graph in Figure 23, the information about the evolution of latency in data writing using the OPC UA protocol fluctuates within the range of 15 to 20 ms. Besides, there are some deviations in latency that vary within the range of 20 to 30 ms. In some cases, latency can be up to 40 ms or even 55 ms. These deviations were observed under the network conditions present during the experiments. Communication is quite stable. The next set of tests was performed using a wireless connection. The graph in Figure 24 depicts the evolution of data latency using the OPC UA protocol for data reading.

Latency during the process of data reading was investigated using the OPC UA communication protocol over a wireless connection, as shown in Figure 24. The latency in most cases was within 50 ms; however, some latency values higher than 500 ms were also found, with the highest value being about 1000 ms. All the variations were observed during the experiments. As the separate components influencing the latencies were not tested separately experimentally, it is impossible to identify the cause for the changes. In the latter part of the measurement period, an increased variability in latency was observed. For the experimental conditions, wireless communication presented low latency in most cases, yet with some variations that include latency spikes of about 1 s. The variability implies that the communication behavior was not temporally deterministic for the tested configuration. For the designed application that performs monitoring and supervisory control, latency variations may affect the update of information and command transmission. There was no observed interference with the operation of the system in the experiment. This makes it possible to use wireless communication for monitoring and supervisory control applications that can accept some response time variations. There was not enough evidence to recommend the tested wireless configuration for control functions that require deterministic timing. Experiments were conducted under normal working conditions of the system, with no additional network load or any special traffic being intentionally introduced. Therefore, the latency variations and high latency values presented in Figure 24 were observed under the normal operating conditions of the system without an artificially introduced network load or specific traffic pattern. These results can be described as the description of the communication efficiency of the tested system, but at the same time, they do not allow us to identify whether network activity or wireless connection causes latency.

To analyze the latency distribution, median, standard deviation, P95, P99, and maximum latency were computed. Such statistical indicators can help to characterize the usual communication performance, its variability, and possible high values. Median and percentile values (P95 and P99) are especially important for the analysis of wireless data because the existence of latency variations and high latency values cannot be estimated using the average value alone. Statistical results of the experiments for all tested scenarios are given in Table 3.

**Table 3:** Statistical analysis of measured communication latency.

Communication Scenario	Median RTT [ms]	SD [ms]	P95 RTT [ms]	P99 RTT [ms]	Maximum RTT [ms]
OPC UA read– Ethernet	14	1.42	16	17	87
Modbus TCP read Ethernet	56	2.99	62	68	130
OPC UA write– Ethernet	14	1.01	15	16	55
OPC UA read Wireless	15	18.86	35	91	1030

## 6. Discussions

In this paper, the aim is to show how it is possible to build a monitoring and supervisory-level control system for an industrial assembly process. This system enables communication with the industrial assembly line as well as the incorporation of data from the industrial machines into a mixed reality (MR) interface. In this paper, we aim to demonstrate the viability and usefulness of mixed reality in an industrial setting, without making any claims of superiority over existing systems. Communication with industrial equipment and data processing in the presented architecture are carried out within one application without a dedicated middleware server. This is a distinctive feature of the suggested architecture, but it does not demonstrate any performance benefits. No experiments comparing architecture with and without middleware have been conducted; thus, no conclusions can be drawn about the influence of removing middleware on performance, usability, or latency. It would be necessary to carry out a controlled experiment using comparable hardware, software, and network environments to draw such conclusions. Differing from the existing applications described in the literature, most mixed reality/augmented reality applications concentrate mainly on providing information about the state of technological processes and instructions on how to use equipment [31,32]. On the contrary, the developed system integrates monitoring functionality with supervisory control functionality. Users may monitor data related to process operation in real time, configure the assembly process, and send commands to the equipment. All the listed functions are provided by means of software scripts and libraries, whereas data exchange is performed via the OPC UA and Modbus TCP protocols within one application. However, comparisons with other solutions need to be viewed in their specific context, since the results might be influenced by differences in system architecture, hardware, network, communication protocols, software implementation, and measurement methodology. In addition, the groups in Table 4, such as SCADA, Web HMI, AR, VR, and MR, involve a wide variety of implementations and architectures. Consequently, Table 4 cannot be viewed as a direct comparison of technology performance, but rather as a comparative analysis of the features and functionality of various solutions.

Experimental results demonstrate the feasibility of implementing MR technology for monitoring industrial processes and issuing supervisory-level commands. The designed interface allows process information to be displayed in the user’s field of view, as well as interaction with the industrial process. These latency measurements reflect the communication properties of the hardware and software configuration used during the experiments and cannot be generalized to other configurations using OPC UA or Modbus TCP. These values were measured under the experimental conditions discussed in this work. Thus, they do not demonstrate deterministic latency in the sense required for industrial systems with strict real-time requirements. In the same way, the data presented in Table 5 reflects the experimental measurements and cannot be treated as minimum or maximum values for the discussed protocols. The differences that have been noted in the results of this experiment compared to those that have been reported in the literature can be attributed to architectural differences in the systems, differences in hardware and software configurations, networking conditions, and measurement techniques. The contribution of each of the above-listed variables towards the observed variations in latency is impossible to isolate experimentally. Thus, the comparison with previous studies is conditional and cannot demonstrate any advantage of the proposed solution. Moreover, there was no comparison with conventional HMI or SCADA systems. Thus, conclusions regarding the usability, speed, or efficiency of the proposed MR interface cannot be drawn, since no comparison with existing HMI/SCADA solutions were made. The contribution of the developed system is described in terms of functionality and architecture and not through any claims about its superiority in terms of performance, usability, or efficiency. Thus, formal user evaluation and a comparison with conventional HMI/SCADA solutions are essential areas for further research. Moreover, an experimental comparison of identical architectures with and without middleware will be needed to understand how this architectural decision affects latency and system behavior. Differences between the proposed system and the types of solutions reviewed in the literature are presented in Table 4.

There are a few limitations associated with the experiment that need to be considered when interpreting the findings. First, the validation of the solution was performed using an assembly line of laboratory scale available within the experimental environment. Thus, the results obtained cannot be generalized to industrial plants of larger scale. Secondly, the results of the wireless communication tests indicated some latency variability in terms of both higher latencies of more than 500 ms and values close to 1 s. Even though such latencies did not affect the proper functioning of supervisory-level monitoring and control tasks within the considered scenario, the wireless communication channel cannot be considered deterministic for time-critical systems. The obtained values of latency reflect the communication characteristics of the experimental setup considered in the study. Lastly, the issue of cybersecurity was not explored in depth. Direct communication with industrial machinery through OPC UA and Modbus TCP must be thoroughly analyzed with respect to such aspects as authentication, authorization, encryption of communication, and prevention of unauthorized access in advance of actual implementation in an industrial setting. Yet another limitation of the research is associated with the lack of a controlled experiment that compares the developed architecture with an equivalent one featuring the middleware server. The findings demonstrate the viability of the direct MR-PLC communication architecture in the selected setting; however, the positive impact of the absence of middleware cannot be proven at this point. The absence of a middleware component from the communication process is a particular feature of the developed architecture. Its impact on system performance would have to be determined with the help of a controlled experiment. This study also does not include any formal user testing. No measurements have been taken of the time that users take to complete tasks, their success rate, and other usability factors. There was also no experiment conducted to compare the new MR HMI/SCADA interface to the traditional interface in terms of ease of use. Thus, while the functionality of the new interface is proven through the implementation of features and the experimental results, it is hard to establish its usability advantage over other interfaces from the current experiment. At the same time, any comparisons to solutions and results mentioned in the literature must be considered in context. Solutions based on SCADA, Web HMI, AR, VR, and MR can have different architectures, hardware, network configurations, communication protocols, and methods of measuring performance. Therefore, the figures obtained in previous experiments are not directly comparable to the figures obtained in the current experiment. Comparisons are made only for the purpose of placing the current results in a broader context of research. Lastly, the scalability of the solution has been examined regarding the setup, protocols, and hardware used in this experiment only. The scalability of the system in an environment where there are many devices, stations, and data streams could demand additional optimization in terms of data processing, communication, and the MR interface. Thus, further experimentation is required.

Table 5 provides a context-based comparison of the proposed system with some of the other systems that use the OPC UA protocol. The communication type, the latency metric reported by them, the presence or absence of middleware, and some observations for each of the cases have been compared here. However, the results from the literature cannot be directly compared with the results from this study due to differences in hardware, software, network, system architecture, configuration, and measurement methods. As a result, the values from the literature are only used for context-based comparison purposes.

For the current study’s experimental setup, the median Round-Trip Time (RTT) for OPC UA write and data reading operations is 14 ms for Ethernet communication. For Modbus TCP communication, the median RTT is 56 ms. This indicates the communication behavior of the experimental setup considered in this study and needs to be considered accordingly. It is difficult to extrapolate the above findings to other OPC UA and Modbus TCP implementations. In the case of OPC UA write operations, even though the median RTT is 14 ms, as shown in Figure 20, there were different levels of response times ranging from approximately 40 to 55 ms. Therefore, the median RTT is merely a statistical description of the communication latency, without suggesting anything about the deterministic nature of the response. The results obtained should not be considered as proof of deterministic latency in the case of OPC UA communication. Another characteristic of the proposed architecture is the lack of an intermediary middleware server for transferring the data. It is an architectural decision and not a performance gain. There is no way to say based on the available experimental data whether the lack of middleware has any impact on the performance or applicability of the system. A controlled experiment using an equivalent architecture with a middleware server would be needed to determine such an impact on the system.

## 7. Conclusions

The system implemented in this research demonstrated that it is possible to use mixed reality (MR) technology to monitor and supervise the operation of the industrial assembly process. The solution presented combines the functionality of collecting and processing information, process visualization, and sending supervisory-level commands to the equipment into a single application. Communication with industrial equipment was provided using OPC UA and Modbus TCP technologies, which allowed the MR application to communicate with the equipment using different communication protocols. Experimental tests confirmed the correctness of the communication with the assembly line and made it possible to characterize the latency and Round-Trip Time (RTT) values for the four examined scenarios. Under the experimental configuration considered, Ethernet-based communication showed lower latency and RTT values compared to the wireless scenario analyzed. Wireless-based communication had high latency variability, including values greater than 500 ms. These findings are indicative of the communication behavior observed in the experimental settings and cannot be used to formulate deterministic communication behavior or ensure performance in applications requiring stringent timing characteristics. The developed MR interface allowed for the visualization of process data, configuration of the assembly’s operating mode, as well as the issuance of supervisory commands to the industrial hardware. Integration of both OPC UA and Modbus TCP protocols in one application demonstrated the feasibility of implementing an MR interface to acquire data and send supervisory commands in an industrial setting with industrial hardware that uses different communication protocols. Nevertheless, these findings should be considered in the context of the experimental setup. Several limitations must be mentioned. The presented system has been tested on an assembly line under laboratory conditions, and it has not been verified on a larger-scale production line in an industrial setting. Moreover, there has been no user testing or experimental comparison of the system with standard HMI/SCADA systems. Therefore, the present results do not prove that the presented MR solution is better than the standard interfaces in terms of usability, intuitiveness, and performance. Cybersecurity techniques have also not been tested experimentally, and the scalability issue has not been studied with a larger number of devices/stations/data streams. Moreover, no experiments have been performed to compare the performance of architecture with and without a middleware server; therefore, there is no evidence of performance improvement resulting from the absence of a middleware server. The future directions of research would be the development of the system to work with a bigger number of industrial devices and communication protocols, as well as the testing of the scalability of the system in different configurations. The research should also be extended to the integration and testing of cybersecurity mechanisms, and to conducting formal user tests comparing the proposed MR solution with traditional HMI/SCADA systems. With the help of such tests, an objective evaluation of the usability and interaction of the proposed solution could be conducted. Also, the integration of mechanisms for predictive maintenance based on the collected process data could be researched.

## Figures and Tables

**Table 1:** Comparison of the analyzed XR systems.

Reference	XR Technology	Industrial Application	Communication Protocols	Direct MR–PLC Communication	Intermediary in MR–PLC Path	Bidirectional Communication	Monitoring	Supervisory Command Transmission
[37]	MR/AR	Industrial process	OPC UA	No	Yes	—	Yes	Limited
[38]	MR/AR	Industrial process	OPC UA	No	Yes	—	Yes	Yes
[39]	MR/AR	Industrial process	OPC UA, MQTT	No	Yes	—	Yes	No
[40]	MR/AR	Industrial process	OPC UA, MQTT, HTTP	No	Yes	—	Yes	Yes
[41]	MR/AR	Industrial process	HTTP	No	Yes	—	Yes	No
[42]	MR/AR	Industrial process	Modbus	No	Yes	Yes	Yes	No
**This work**	**MR**	**Assembly line**	**OPC UA, Modbus TCP**	**Yes**	**No**	**Yes**	**Yes**	**Yes**

**Table 2:** Changes in active power during operating cycles.

Act. Pwr	M1	M2	M3	M4	M5	M6	M7
**Min**	11.90014935	39.32847977	171.1553955	177.9447327	221.0571	230.2269	259.3848
**Max**	43.32278061	46.16279221	1147.422607	189.8589935	254.6905	1171.96	422.4926
**Avg**	24.6319269	45.34749056	432.269751	187.3699748	243.7813	335.2165	311.4204
**Med**	24.88558769	45.477222626	173.1761627	186.8445435	244.2919	264.7806	317.4741
**SD**	1.514783235	0.688163975	376.4748148	1.386525857	2.757777	186.3583	41.14515
**Var**	2.294568249	0.473569657	141,733.2862	1.922453952	7.605334	34729.43	1692.923

**Table 4:** Comparison of industrial monitoring and control systems.

System Type	Direct PLC Communication	Integrated Data Processing	Dedicated External Server	Multiple Industrial Protocols	Process Monitoring	Supervisory Bidirectional Control	Technology
SCADA	Depends on architecture	Depends on architecture	Depends on architecture	Yes	Yes	Yes	2D
Web HMI	Depends on architecture	Depends on architecture	Usually required	Partial	Yes	Limited	2D
AR maintenance	Depends on implementation	Yes	Depends on implementation	Depends on implementation	Partial	No	AR
VR training	No	Yes	No	No	No	No	VR
MR visualization	Depends on implementation	Depends on implementation	Depends on implementation	Partial	Yes	No	MR
**Proposed system**	**Yes**	**Yes**	**No**	**Yes**	**Yes**	**Yes**	**MR**

**Table 5:** Comparison of communication performance with existing systems.

Reference	Communication	Latency Metric	Measured Latency	Middleware	Remarks
[35]	OPC UA client-server	RTT	20–50 ms	No	Protocol benchmark
[36]	OPC UA PubSub	RTT	40–80 ms	Yes	Cloud communication
[37]	OPC UA over TSN	Latency and jitter	N/A	No	TSN evaluation
[38]	OPC UA over TSN	Latency	N/A	Yes	Fog architecture
This work	OPC UA Read (Ethernet)	Median RTT	14 ms	No	Direct MR communication
This work	OPC UA Write (Ethernet)	Median RTT	14 ms	No	Low median RTT
This work	Modbus TCP (Ethernet)	Median RTT	56 ms	No	Stable communication
This work	OPC UA Read (Wi-Fi)	Median RTT	15 ms	No	Wireless communication

## Data Availability

The experimental data employed in this study was collected through experimentation on an industrial assembly line set up in an industrial laboratory. The experimental data analyzed and summary statistics are presented in this paper. The full raw experimental data is obtainable from the corresponding author on request.

## Abbreviations

The following abbreviations are used in this manuscript:

OPC	Open platform communication
TCP	Transmission Control Protocol
HMI	Human–Machine Interface
SCADA	Supervisory Control and Data Acquisition
IoT	Internet of Things
MR	Mixed Reality
MQTT	Message Queuing Telemetry Transport
HTTP	Hypertext Transfer Protocol
DT	Digital Twin
PDM	Product Data Management
TOS	Terminal Operating System
ECS	Equipment Control System
DB	Database
RFID	Radio Frequency Identification
MRTK	Mixed Reality Toolkit
IP	Internet Protocol
VR	Virtual Reality
TSN	Time-Sensitive Networking
RTT	Round-Trip Time
